# Mobility status, nutritional intervention and meal eaten are associated with discharge home: the nutritionDay study in China

**DOI:** 10.3389/fnut.2025.1631276

**Published:** 2025-09-23

**Authors:** Bei Zhou, Yupeng Zhang, Xuejin Gao, Sitong Liu, Ruting Shen, Yingchun Huang, Yang Zhao, Li Zhang, Xinying Wang

**Affiliations:** ^1^Clinical Nutrition Service Center, Department of General Surgery, Jinling Clinical Medical College, Nanjing University of Chinese Medicine, Nanjing, China; ^2^School of Health Preservation and Rehabilitation, Nanjing University of Chinese Medicine, Nanjing, China; ^3^Clinical Nutrition Service Center, Department of General Surgery, Jinling Hospital, Medical School of Nanjing University, Nanjing, China; ^4^Department of Biostatistics, School of Public Health, Nanjing Medical University, Nanjing, China

**Keywords:** mobility status, discharge home, nutritional intervention, meal eaten, nutritionDay

## Abstract

**Objectives:**

Reduced mobility during hospitalization can trigger complications and worsen the prognosis of critically ill patients. However, evidence regarding the combined effects of mobility status and nutritional characteristics on inpatients is scarce. This study aimed to evaluate how inpatients’ mobility status, nutritional intervention, and meal eaten affect discharge home and to further explore the relationship between reduced mobility and nutritional characteristics.

**Methods:**

This was a prospective cross-sectional nutritionDay study conducted at 20 centers in China from 2010 to 2020. The sample was divided into mobile and reduced mobility groups. Cox regression models were used to identify the potential effects of nutritional intervention, meal eaten, and mobility status on discharge home. Logistic regression models were used to identify the association between reduced mobility and nutritional characteristics. Subgroup analyses were conducted to evaluate the association between mobility status and discharge home according to different nutritional interventions, meal eaten, survey years, and regions. Sensitivity analyses were performed to assess the robustness of the findings.

**Results:**

A total of 5,511 adult patients were included. Mobile patients who could walk unaided had a 1.2 to 1.3 times higher chance of discharge home and a significantly shorter duration of hospital stay after nutritionDay than those with reduced mobility, especially in patients receiving artificial nutrition (median: 8 days vs. 11 days, *p* < 0.001) and those with incomplete meal intake (median: 6 days vs. 8 days, *p* < 0.001). In multivariable models accounting for other parameters, patients receiving dietary nutrition had a 0.45 (95% CI [0.36–0.55], *p* < 0.001) lower probability of reduced mobility compared with patients receiving artificial nutrition, and patients who ate their meals completely had a 0.34 (95% CI [0.28–0.41], *p* < 0.001) decreased chance of reduced mobility compared with those with incomplete meal intake.

**Conclusion:**

Walking unaided was associated with a higher chance of discharge home, particularly in patients receiving artificial nutrition and those with incomplete meal intake. The likelihood of reduced mobility can be estimated by nutritional intervention and meal eaten on the same day. Further studies are required to validate causal inference and improve inpatient mobilization by addressing relevant influencing factors.

## 1 Introduction

Hospital mobility is defined as physical activity around the ward (1), which is vital to the care of hospitalized patients (2). Reduced mobility can trigger complications and worsen the prognosis of critically ill patients (3, 4). However, the epidemic of reduced mobility has been spreading probably due to efforts to avoid the risk of falls (1, 5, 6) and worsened nutritional status (7). Assistance with walking and eating has been associated with lower nutritional intake (7), leading to restrictions in life independence during hospitalization.

As a worldwide scientific audit concerning nutritional care and mobility status in hospitalized patients, nutritionDay is a cross-sectional study followed by a 30-day clinical outcome evaluation, supported by the European Society of Clinical Nutrition and Metabolism (ESPEN) (8). The first nutritionDay survey was conducted in 2006 (9). To date, the nutritionDay team has been promoting participation in hospital units and nursing homes, as well as updating the structured questionnaires since 2016 to optimize large-scale real-world studies (10).

Despite adopting a version of nutritionDay 2.0 based on the 2006–2015 questionnaires (10), information on nutritional intervention, meal eaten, and mobility on the survey days has been consistently collected and has received attention from researchers. Previous studies have indicated that patients receiving artificial nutrition had a prolonged length of hospital stay (LOS) (11, 12), whereas food provision was related to improved outcomes (13). Less meal consumption was associated with a higher risk of mortality within 30 days compared with entire meal intake (14). Moreover, decreased eating was also associated with decreased mobility (15). However, it remains unclear to what extent nutritional intervention and meal eaten affect mobility status. Additionally, the relationship between mobility status, nutritional characteristics, and clinical outcomes needs further exploration.

Considering that patients with impaired mobility had a more prolonged mean LOS than patients walking unaided (15), we aimed to: (1) evaluate how inpatients’ mobility status combined with nutritional intervention and meal eaten affect clinical outcomes; and (2) explore the relationship between reduced mobility and nutritional characteristics based on the nutritionDay survey from 2010 to 2020 in China.

## 2 Materials and methods

### 2.1 Study population

This was a prospective cross-sectional cohort study with 20 centers located around the east, south, north, and northwest regions in China from 2010 to 2020 (13). All subjects provided informed consent and were assured the right to refuse participation at any time. The nutritionDay study was approved by the Ethics Committee of the Medical University of Vienna (EK407/2005) and the Ethics Committee of Jinling Hospital, the Chinese host hospital, with annual amendments (13). In the present study, 310 patients without data on mobility status were excluded from 2010-2020 cohort (13).

### 2.2 Data collection and classification

Hospital questionnaires are freely available in different languages worldwide on the nutritionDay website (16). Patients’ general characteristics, including nutritional history and care data, were collected through these questionnaires divided into four sheets. Sheet 1 recorded structural information about the participating unit and hospital. Sheet 2 collected disease-related and nutritional information for each patient, recorded by unit staff. Sheet 3 contained self-reported general status and current conditions completed by patients themselves. Sheet 4 collected patients’ clinical outcomes and discharge dates.

Nutritional intervention, meal eaten, and mobility status on each survey day were tracked using validated standardized questionnaires (10, 16). In Sheet 2, unit staff recorded patients’ nutritional interventions, including hospital food, special diets, protein supplements, enteral nutrition, and parenteral nutrition. In Sheet 3, patients indicated the quantity of meal eaten on nutritionDay as “all,” “1/2,” “1/4,” or “nothing.” Patients also reported their actual mobility status by answering the question “Can you walk without assistance today?” with options “yes,” “no, only with assistance,” or “no, I stay in bed.” For outcome analysis, nutritional intervention was classified as none, artificial nutrition (including protein/energy supplements, enteral nutrition, parenteral nutrition, and multi-form of artificial nutrition), dietary nutrition (including regular hospital food, fortified/enriched hospital food, special diet, and multiple forms of dietary nutrition), and multi-form of artificial and dietary nutrition. Meal eaten was dichotomized as completely eaten or incompletely eaten (including half, quarter or no meal eaten). Mobility status was categorized as mobile (walking without assistance) and reduced mobility (including walking with assistance and bedridden).

### 2.3 Outcomes

Patients’ clinical outcomes were reassessed 30 days after nutritionDay by unit staff. According to outcome codes and discharge dates, outcomes were categorized as discharged home, still in hospital, transferred and death (15). In the present study, discharge home was the primary outcome. The association between reduced mobility and nutritional characteristics was also analyzed.

### 2.4 Statistical analysis

Demographic characteristics and clinical information are presented as median with interquartile range (IQR) for continuous variables, and counts with percentages for categorical variables. Continuous variables such as age, LOS since admission, and body mass index (BMI) were classified into categories. To enable comparison with previous studies (13, 17, 18), cutoffs were chosen according to 10-year age groups, median days since admission, and the World Health Organization classification of BMI (19), respectively. Missing values were treated as a separate category. Comparisons between patient groups based on mobility status were performed using the Chi-square test, Fisher’s exact test, or Wilcoxon rank-sum test, as appropriate. Cox regression models were used to identify the potential effects of nutritional intervention, meal eaten, and mobility status on discharge home within 30 days after nutritionDay. The validity of results was assessed with covariates including departments, survey year, hospital location, sex, BMI, weight change within the last 3 months, major lesion types, comorbidities, food intake in the previous week, previous intensive care unit stay, self-rated health, surgical status, LOS before nutritionDay, and number of drugs before admission across three multivariable models: Model I included nutritional intervention, meal eaten and mobility status; Model II included meal eaten and combined nutritional intervention with mobility status; and Model III included nutritional intervention and combined meal eaten with mobility status. Cumulative incidence curves of discharge home were plotted by mobility status (mobile vs. reduced mobility) for all patients, patients with different nutritional interventions (artificial nutrition vs. dietary nutrition) and patients with different meal eaten (completely eaten vs. incompletely eaten). Differences between groups were compared by log-rank tests. Logistic regression models identified the association between reduced mobility and patients’ characteristics. Odds ratios (ORs), hazard ratios (HRs) or medians were reported with 95% confidence intervals (CIs).

Cox regression subgroup analyses of discharge home were conducted based on patients with artificial nutrition, dietary nutrition, complete meal eaten, and incomplete meal eaten on nutritionDay, respectively. As nutritionDay was initiated across western and eastern regions of China since 2016, the main analysis was also repeated separately according to survey years (before and after 2016) and regions (eastern and western).

Additionally, four sensitivity analyses assessed the robustness of findings: (1) analyses excluding missing values; (2) analyses limited to patients with digestive disease as the primary diagnosis; (3) analyses adjusted for pre-hospital functional status including weight change within the last 3 months, food intake in the previous week, and number of drugs before admission; (4) analyses adjusted for underlying comorbidities.

All statistical analyses were performed using R version 4.2.1. A *p*-value < 0.05 was considered statistically significant.

## 3 Results

### 3.1 Demographic and nutritional characteristics of the inpatients

A total of 5,511 patients were included in this study, 59.4% of whom were male, with an average age of 58 years (IQR 45–68) and an average BMI of 22.8 kg/m^2^ (IQR 20.2–25.2). About one of every three patients was postoperative. On nutritionDay, more than half of the patients were provided with dietary nutrition. However, 59% of patients did not complete their meals. A total of 23.2% of patients who could not walk without assistance were defined as having reduced mobility. Notably, patients with reduced mobility received artificial nutrition more frequently: 19.2% of mobile patients were provided artificial nutrition, compared with 36.5% of patients with reduced mobility. In contrast, 56.5% of mobile patients received dietary nutrition, compared with 37.8% of patients with reduced mobility. Regarding food intake, 43.3% of mobile patients ate a full meal, which decreased to 19.9% among patients with reduced mobility. Moreover, 75.6% of reduced mobility patients did not complete a full meal, with a particularly high proportion of patients eating nothing (38.5%). During the 30 days after nutritionDay, 4,809 patients (87.3%) were discharged home. However, a significantly higher proportion of postoperative status, longer LOS before nutritionDay, and lower prevalence of discharge home were observed in reduced mobility patients (Table 1).

**Table 1:** Demographic and nutritional characteristics of patients according to mobility status.

Variable	All, *n* (%)	Mobile, *n* (%)	Reduced mobility, *n* (%)	*p-*value
	5,511 (100%)	4,232 (76.8%)	1,279 (23.2%)	
General characteristics				
Sex, %, female/male	40.5/59.4	40.3/59.7	41.2/58.7	0.814
Age, y, median (IQR)	58.0 (45.0–68.0)	56.0 (45.0–66.3)	61.0 (48.0–71.0)	< 0.001
BMI, kg/m^2^, median (IQR)	22.8 (20.2–25.2)	22.9 (20.4–25.3)	22.4 (19.8–24.8)	< 0.001
Postoperative, *n* (%)	1702 (30.9%)	1,143 (27.0%)	559 (43.7%)	< 0.001
LOS before nutritionDay, days, median (IQR)	6.0 (3.0–12.0)	5.0 (2.0–10.0)	9.0 (4.0–16.0)	< 0.001
Food intake in the previous week, *n* (%)				< 0.001
More than normal or normal	3,661 (66.4%)	3,002 (70.9%)	659 (51.5%)	
A little less than normal	707 (12.8%)	492 (11.6%)	215 (16.8%)	
Less than half of normal	1,104 (20.0%)	706 (16.7%)	398 (31.1%)	
Missing	39 (0.7%)	32 (0.8%)	7 (0.5%)	
Nutritional intervention, *n* (%)				< 0.001
Artificial nutrition	1,280 (23.2%)	813 (19.2%)	467 (36.5%)	
Dietary nutrition	2,877 (52.2%)	2,393 (56.5%)	484 (37.8%)	
Multi-form of artificial and dietary nutrition	569 (10.3%)	408 (9.6%)	161 (12.6%)	
None	175 (3.2%)	137 (3.2%)	38 (3.0%)	
Unsure or missing	610 (11.1%)	481 (11.4%)	129 (10.1%)	
Eating on nutritionDay, *n* (%)				< 0.001
Eaten all	2087 (37.9%)	1832 (43.3%)	255 (19.9%)	
Eaten half	1,143 (20.7%)	860 (20.3%)	283 (22.1%)	
Eaten quarter	534 (9.7%)	343 (8.1%)	191 (14.9%)	
Eaten nothing	1,575 (28.6%)	1,082 (25.6%)	493 (38.5%)	
Missing	172 (3.1%)	115 (2.7%)	57 (4.5%)	
Outcome, *n* (%)				< 0.001
Discharged home	4,809 (87.3%)	3,792 (89.6%)	1,017 (79.5%)	
Still in hospital	369 (6.7%)	227 (5.4%)	142 (11.1%)	
Transferred	205 (3.7%)	127 (3.0%)	78 (6.1%)	
Death	27 (0.5%)	11 (0.3%)	16 (1.3%)	
Unsure or missing	101 (1.8%)	75 (1.8%)	26 (2.0%)	

BMI, body mass index; IQR, interquartile range; LOS, length of hospital stay. Variables are shown as median and IQR (25th–75th percentiles) according to the distribution; categorical variables are shown as number (%) and compared by Chi-square test, Fisher’s exact test, or Wilcoxon rank-sum test appropriately between mobile patients and reduced mobility patients.

### 3.2 Nutritional intervention, meal eaten and 30-day outcomes between patients with different mobility status

**Figure 1:**
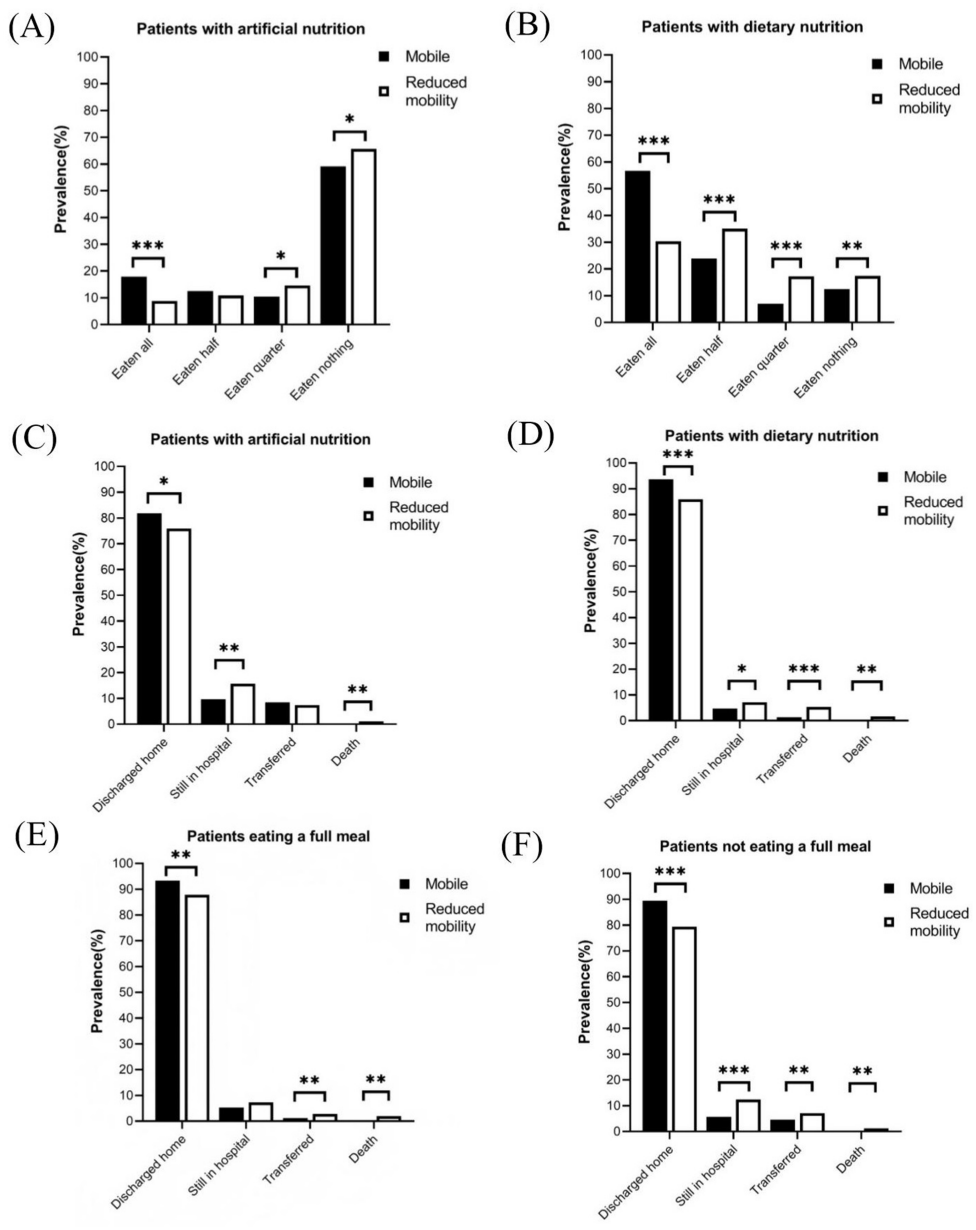
Meal eaten and discharged home in patients with different nutritional interventions or meal eaten on nutritionDay. **(A)** Meal eaten in patients with artificial nutrition. **(B)** Meal eaten in patients with dietary nutrition. **(C)** Clinical outcomes in patients with artificial nutrition. **(D)** Clinical outcomes in patients with dietary nutrition. **(E)** Clinical outcomes in patients with completely eaten. **(F)** Clinical outcomes in patients with incompletely eaten. Comparisons between patient groups by mobility status were performed using the Chi-square test, Fisher’s exact test, or Wilcoxon rank-sum test, as appropriate. *p < 0.05; **p < 0.01; ***p < 0.001.

Different mobility status regarding nutritional intervention, meal eaten, and 30-day clinical outcomes according to mobility status are shown in Figure 1. Among patients receiving artificial nutrition (Figure 1A), mobile patients had a higher probability of eating all (17.9% vs. 8.8%, *p* < 0.001) and a lower probability of eating nothing (59.1% vs. 65.7%, *p* < 0.05) than those with reduced mobility. Among patients receiving dietary nutrition, those with mobile status demonstrated a significantly higher rate of eating all than those with reduced mobility (56.6% vs. 30.3%, *p* < 0.001, Figure 1B). Furthermore, the proportion of patients discharged home was significantly higher among mobile patients than those with reduced mobility (Figures 1C–F). Specifically, among patients receiving dietary nutrition, 93.7% of mobile patients and 85.9% of reduced mobility patients were discharged home within 30 days after nutritionDay (*p* < 0.001, Figure 1D). Similar results were observed in patients who ate a full meal (93.3% vs. 87.9%, *p* < 0.01, Figure 1E). Even among patients receiving artificial nutrition, 81.8% of mobile patients were discharged home within 30 days, compared with 75.9% of patients with reduced mobility (*p* < 0.05, Figure 1C).

### 3.3 Mobility, nutritional intervention and meal eaten associated with discharged home

**Figure 2:**
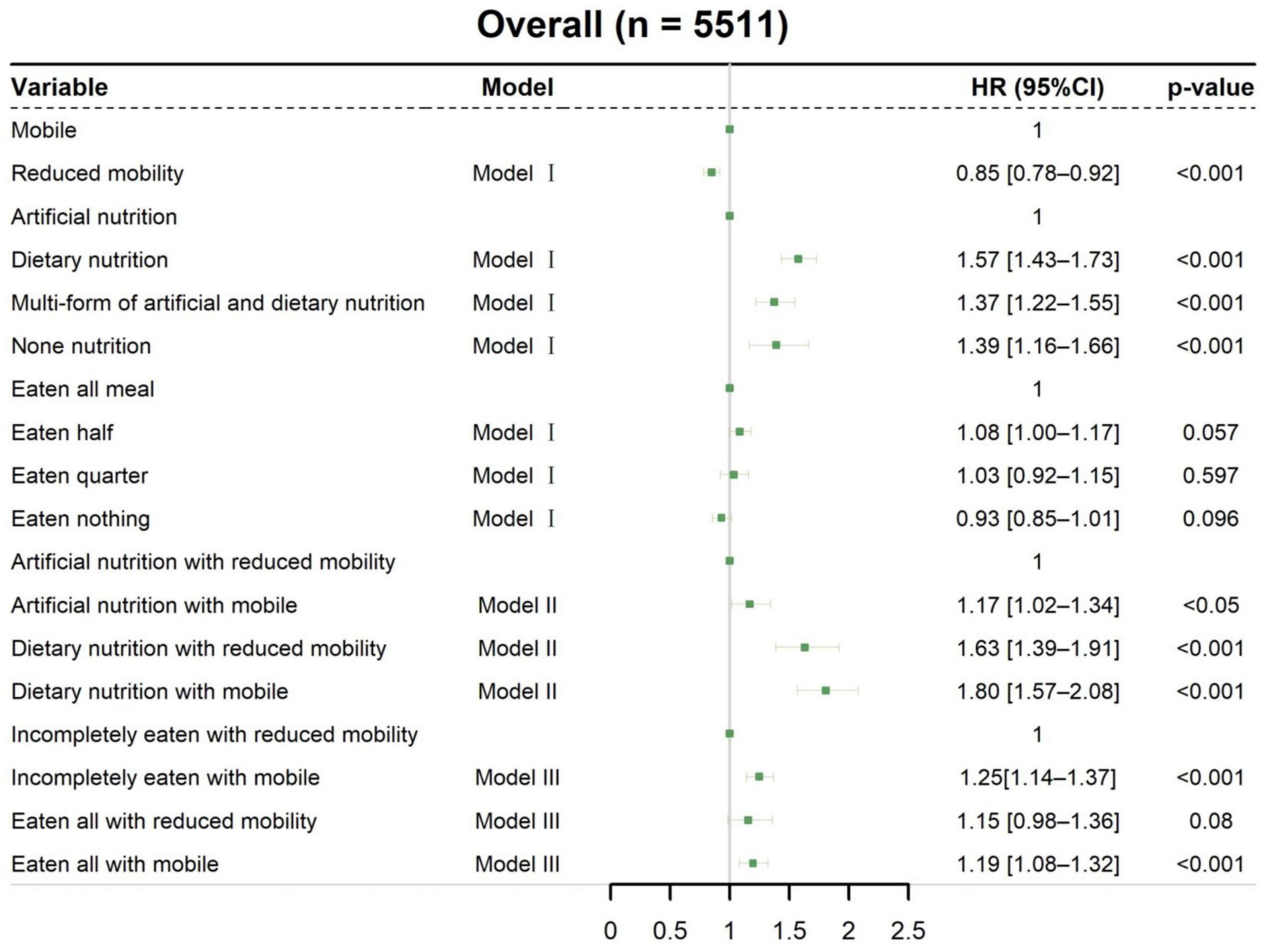
Associations of mobility, nutritional intervention, and meal eaten with discharged home. Cox regression models with HRs were used to analyze discharged home. Model I: Multivariable analysis including nutritional intervention, meal eaten, and mobility status. Model II: Meal eaten and combined nutritional intervention with mobility status added to the multivariable analysis. Model III: Nutritional intervention and combined meal eaten with mobility status added to the multivariable analysis. All data are presented as HR and 95% CI. HR, hazard ratio; CI, confidence interval.

Cox regression models were used to determine the effects of mobility, nutritional intervention, and meal eaten on discharge home within 30 days after nutritionDay (Figure 2). In univariate analysis (Supplementary Table S1), the HR for discharge home was 0.70 (95% CI [0.66–0.75], *p* < 0.001) for reduced mobility patients compared with mobile patients. Similar trends were found in the multivariate analysis of Model I, which showed that reduced mobility patients had a decreased probability of discharge home (HR 0.85, 95% CI [0.78–0.92], *p* < 0.001, Figure 2). The association between mobility status and discharge home is also visualized by cumulative incidence curves (Supplementary Figure S1C). It should be noted that mobile patients had a median LOS of 6 days (IQR 6–6) after nutritionDay, whereas reduced mobility patients had a median LOS of 8 days (IQR 8–9) after nutritionDay (*p* < 0.001), indicating a better prognosis for patients who can walk without assistance.

**Figure 3:**
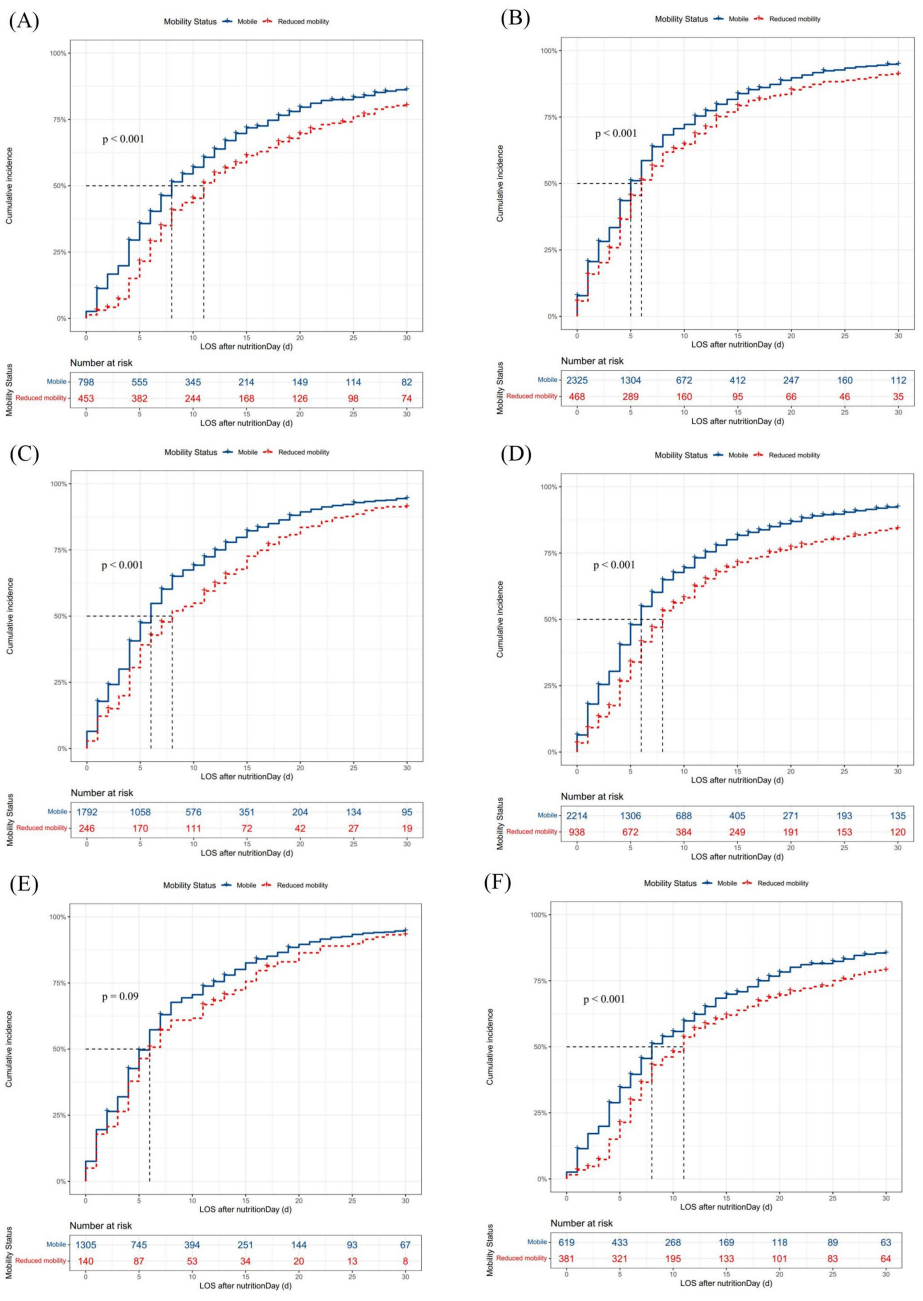
Cumulative incidence of discharged home within 30 days after nutritionDay in patients with different nutritional interventions and meal eaten on the survey days. Missing data were excluded. Differences in median (95% CI) LOS after nutritionDay between groups were tested using the log-rank test. LOS, length of hospital stay; CI, confidence interval. **(A)** Mobility status in patients with artificial nutrition, *n* = 1,280. Mobile vs. reduced mobility: 8 days (8–9) vs. 11 days (11–13), *p* < 0.001. **(B)** Mobility status in patients with dietary nutrition, *n* = 2,877. Mobile vs. reduced mobility: 5 days (5–6) vs. 6 days (6–7), *p* < 0.001. **(C)** Mobility status in patients with completely eaten, *n* = 2087. Mobile vs. reduced mobility: 6 days (6–6) vs. 8 days (7–11), *p* < 0.001. **(D)** Mobility status in patients with incompletely eaten, *n* = 3,252. Mobile vs. reduced mobility: 6 days (5–6) vs. 8 days (7–9), *p* < 0.001. **(E)** Mobility status in patients with dietary nutrition and completely eaten, *n* = 1,480. Mobile vs. reduced mobility: 6 days (5–6) vs. 6 days (5–8), *p* = 0.09. **(F)** Mobility status in patients with artificial nutrition and incompletely eaten, *n* = 1,022. Mobile vs. reduced mobility: 8 days (8–10) vs. 11 days (9–12), *p* < 0.001.

When combining nutritional intervention with mobility status on nutritionDay in Model II (Figure 2), mobile patients with artificial nutrition (HR 1.17, 95% CI [1.02–1.34], *p* < 0.05) and dietary nutrition (HR 1.80, 95% CI [1.57–2.08], *p* < 0.001), as well as reduced mobility patients with dietary nutrition (HR 1.63, 95% CI [1.39–1.91], *p* < 0.001), had an increased probability of discharge home compared with reduced mobility patients with artificial nutrition. Thus, clinical staff need to pay more attention to mobility status, particularly in patients with artificial nutrition, as these patients with reduced mobility had a significantly prolonged median LOS after nutritionDay compared with mobile patients (11 days vs. 8 days, *p* < 0.001, Figure 3A). Among patients receiving dietary nutrition, those who could walk without assistance had a significantly shorter median LOS after nutritionDay compared with patients with reduced mobility (5 days vs. 6 days, *p* < 0.001, Figure 3B). Similarly, when combining meal eaten and mobility status in Model III (Figure 2), compared with reduced mobility patients who did not completely eat on nutritionDay, the HRs for discharge home were 1.19 (95% CI [1.08–1.32], *p* < 0.001) and 1.25 (95% CI [1.14–1.37], *p* < 0.001) for mobile patients with and without a full meal eaten, respectively. Similar clinical phenomena were observed in these patients regardless of meal eaten (Figures 3C,D), showing that patients with reduced mobility had a significantly prolonged median LOS after nutritionDay compared with mobile patients (8 days vs. 6 days, *p* < 0.001). Strikingly, there was no significant difference in discharged home (Supplementary Figure S2) and median LOS after nutritionDay between mobile and reduced mobility patients in those receiving dietary nutrition and having eaten completely (6 days vs. 6 days, *p* = 0.09, Figure 3E). In contrast, mobile patients had nearly a 1.3-fold higher chance of discharge home (Supplementary Figure S3) and a shortened median LOS after nutritionDay compared with reduced mobility patients, particularly those both receiving artificial nutrition and incompletely eating (8 days vs. 11 days, *p* < 0.001, Figure 3F).

The same variables remained in models stratified by survey years and regions in subgroup analyses, with estimates similar to the original models (Supplementary Figures S4–S7). In subgroup analyses of patients with different nutritional interventions and varying meal intake (Supplementary Figures S8–S11), patients with reduced mobility had a significantly decreased probability of discharge home when receiving artificial nutrition (HR 0.82, 95% CI [0.70–0.95], *p* < 0.01, Supplementary Figure S8) and when incompletely eating (HR 0.79, 95% CI [0.72–0.87], *p* < 0.001, Supplementary Figure S11). Particularly in patients with artificial nutrition or incompletely eaten, patients with mobile status had a 1.2 to 1.3-fold higher chance of discharge home than those with reduced mobility, consistent with the original model (Supplementary Figures S8, S11).

Sensitivity analyses based on digestive disease as the primary diagnosis also showed that patients with reduced mobility had a significantly decreased probability of discharge home within 30 days compared with mobile patients (HR 0.79, 95% CI [0.69–0.90], *p* < 0.001, Supplementary Figure S12). Especially in patients with digestive disease receiving artificial nutrition, mobile patients had a significantly increased probability of discharge home (HR 1.31, 95% CI [1.07–1.61], *p* < 0.01, Supplementary Figure S12) and a shortened median LOS after nutritionDay (9 days vs. 13 days, *p* < 0.01, Supplementary Figure S16E) compared with reduced mobility patients. Similarly, among digestive disease patients who did not eat a full meal on nutritionDay, mobile patients had a 1.35-fold higher chance of discharge home (95% CI [1.17–1.57], *p* < 0.001, Supplementary Figure S12) and a 3-day shorter median LOS after nutritionDay compared with reduced mobility patients (6 days vs. 9 days, *p* < 0.001, Supplementary Figure S16F). Findings from other sensitivity analyses, which excluded missing values, adjusted for pre-hospital functional status, and adjusted for underlying comorbidities, were consistent with the main analyses (Supplementary Figures S13–S16).

### 3.4 Nutritional characteristics of patients with mobility status on nutritionDay

The association between reduced mobility and patients’ characteristics was analyzed by logistic regression. Univariate analysis (Supplementary Table S2) showed that patients reporting weight loss within the last 3 months and food intake less than normal in the previous week were more likely to have reduced mobility, indicating that weight loss and reduced food intake should be noted in patients with reduced mobility. In multivariable analysis (Table 2), patients receiving dietary nutrition were less likely to have reduced mobility compared with patients receiving artificial nutrition (OR 0.45, 95% CI [0.36–0.55], *p* < 0.001), and patients who ate completely had a lower odds of reduced mobility than those who ate incompletely (OR 0.34, 95% CI [0.28–0.41], *p* < 0.001). Similar estimates were observed in sensitivity analyses (Supplementary Tables S3–S6), underscoring the importance of monitoring nutritional intervention and meal intake in patients with reduced mobility during hospitalization.

**Table 2:** Nutritional characteristics of patients with reduced mobility on nutritionDay.

Variable	Category	Univariate analysis	Multivariable analysis
		OR [95% CI]	OR [95% CI]
Weight change within last 3 months	Stable weight	Reference	Reference
	Weight loss	1.82 [1.58–2.09] ^***^	1.14 [0.95–1.36]
	Weight gain	0.86 [0.63–1.19]	0.92 [0.64–1.32]
	Unsure or missing	2.00 [1.62–2.47] ^***^	1.53 [1.18–1.98] ^**^
Food intake in the previous week	More than normal or normal	Reference	Reference
	A little less than normal	1.99 [1.66–2.39] ^***^	1.08 [0.86–1.36]
	Less than half of normal	2.57 [2.21–2.98] ^***^	1.34 [1.10–1.64] ^**^
	Missing	1.00 [0.44–2.27]	0.61 [0.25–1.53]
Nutritional intervention	Artificial nutrition	Reference	Reference
	Dietary nutrition	0.35 [0.30–0.41] ^***^	0.45 [0.36–0.55] ^***^
	Multi-form of artificial and dietary nutrition	0.69 [0.55–0.85] ^***^	0.70 [0.54–0.92] ^**^
	None	0.48 [0.33–0.70] ^***^	0.90 [0.58–1.39]
	Unsure or missing	0.47 [0.37–0.58] ^***^	0.64 [0.48–0.84] ^**^
Eating on nutritionDay	Incompletely eaten	Reference	Reference
	Completely eaten	0.33 [0.28–0.38] ^***^	0.34 [0.28–0.41] ^***^
	Missing	1.17 [0.85–1.62]	0.91 [0.61–1.37]

OR, odds ratio; CI, confidence interval. For reduced mobility, logistic regression models with ORs were conducted. *p < 0.05, **p < 0.01, ***p < 0.001.

## 4 Discussion

In this multicenter cross-sectional study of the nutritionDay China cohort, we explored the effects of inpatients’ mobility status combined with nutritional intervention and meal eaten on discharge home within 30 days, and examined to what extent nutritional intervention and meal eaten affect mobility status. First, mobile patients had a higher chance of discharge home and a shortened LOS after nutritionDay than those with reduced mobility, especially in patients receiving artificial nutrition and those who ate incompletely. Second, patients with reduced mobility less frequently ate a complete meal on nutritionDay. Third, patients receiving dietary nutrition had a 0.5 lower probability of reporting reduced mobility compared with patients receiving artificial nutrition, and patients who ate completely had a 0.3 lower probability of reduced mobility than those who ate incompletely in multivariable models accounting for other parameters.

On nutritionDay, less than 20% of mobile patients who received artificial nutrition ate a full meal, which dropped by more than half in the reduced mobility patients. In contrast, among patients receiving dietary nutrition, one third of reduced mobility patients finished their meals, increasing to more than half in mobile patients. With reduced mobility, the frequency of eating nothing was markedly higher in patients receiving artificial nutrition. Moreover, the highest and lowest rates of discharge home within 30 days were observed in mobile patients with dietary nutrition and reduced mobility patients with artificial nutrition, respectively. A prospective cohort study conducted in community-dwelling older adults showed that lower protein intake was associated with a higher chance of mobility limitation (20). Considering that impaired food intake is associated with immobility in the ward (15), the combined effects of mobility status with nutritional intervention or meal eaten on clinical outcomes warrant attention in this study.

The negative relationship between reduced mobility and discharge home was demonstrated in both univariate and multivariable models. Patients with reduced mobility had a 0.85 lower chance of discharge home compared with mobile patients in multivariable analysis, consistent with Latin America nutritionDay results showing that inability to walk alone was a significant risk factor affecting mortality (21). Notably, reduced mobility, leading to loss of independence, negatively impacted clinical outcomes and post-discharge quality of life (1, 22), while improved mobility was associated with better quality of life in patients (23).

In the present study, mobile patients had a 1.2 to 1.3 times higher chance of discharge home compared with reduced mobility patients among those receiving artificial nutrition and who ate incompletely. Even in sensitivity analysis based on digestive disease as the primary diagnosis, mobile patients had a 1.3 times higher probability of discharge home compared with reduced mobility patients. These findings highlight the urgency of monitoring mobility status in patients receiving nutritional support and suffering from digestive disease. A March 2025 review by González-Seguel et al. suggested that the combination of nutritional intervention and physical rehabilitation showed synergistic benefits in critically ill patients based on evidence published between 2023 and 2024 (24). Abe et al. retrospectively demonstrated that adequate nutrition combined with early mobilization increased skeletal muscle area in septic patients (25), whereas Silva-Gutiérrez et al. prospectively found that severe and critically ill COVID-19 patients experienced reductions in muscle mass and mobility during hospitalization (26). As reduced mobility intrinsically reflects disease severity and frailty (25–27), mobility status could act as both an independent predictor and a proxy for unmeasured severity. Although patients with poor muscle quality had a higher probability of mobility problems (27), a randomized controlled trial in patients with intestinal failure found that supervised resistance training improved sarcopenia when patients received nutritional therapy (28). When combining meal eaten with mobility status, mobile patients also had a significantly higher chance of discharge home with digestive disease and incompletely eaten meals, reflecting a potential positive effect of unaided walking during hospitalization even in digestive disease patients who did not finish their meals. Personal characteristics including age, cognitive and functional status, as well as LOS, have been shown to be associated with mobility status and food intake (18, 29). Moreover, daily protein intake and physical activity are both necessary for maintaining muscle mass and muscle function (30). Our findings emphasize that mobility status may be a significant issue in patients receiving artificial nutritional support or those with insufficient meal intake. Meanwhile, given that we controlled for other demographic and nutritional parameters, we also propose that encouraging inpatients to try walking independently may play a more active role in prognosis even without optimized nutritional intake.

Regarding discharge home within 30 days after nutritionDay, patients with reduced mobility had a significantly prolonged median LOS of 1 day compared with mobile patients when receiving dietary nutrition. In patients receiving artificial nutrition, reduced mobility patients had a median LOS of 11 days after nutritionDay, which was significantly prolonged by 3 days compared with mobile patients. These findings highlight the importance of mobilization in patients receiving artificial nutritional support. Notably, among patients with digestive disease and artificial nutrition, reduced mobility patients had a median LOS prolonged by 4 days after nutritionDay compared with mobile patients. These results reveal that combining artificial nutrition with reduced mobility is associated with an apparently lower chance of discharge home. Given that low mobility is a sign of frailty during hospitalization (29, 31), standardized hospital-wide mobility training should be planned for patients by clinical staff to improve prognosis (32–34). Núñez-Cortés et al. conducted a randomized controlled trial in patients after total knee arthroplasty and found that patients’ physical function benefited from intensive elastic resistance training (35). Implementation is a call to action to improve mobility status during hospitalization. Especially for patients receiving artificial nutrition, protocols should target specific mobility goals during hospitalization and after discharge (1, 36). Measurements such as the timed-up-and-go test (37), the six-minute walk test (38), and five times sit-to-stand test (39) have been demonstrated as physical performance indicators related to functional independence (40). In clinical settings, hospital mobility is not only limited to walking around hallways but also involves a spectrum of physical activities in the patient’s room, including sitting, standing, and limited activities (1). Strategies encouraging inpatients to move around or walk outside the room are necessary. Furthermore, clinical staff should collaborate with physical therapists to provide a step-by-step scheme for mobility implementation that encompasses daily plan scheduling, mobility training, patient education, and engagement (32, 34). A randomized clinical study by Greysen et al. designed a mobility game for patients using a wearable device to track daily steps and showed that patients with social support partners increased mobility after discharge (41).

Furthermore, 23.2% of hospitalized patients self-reported reduced mobility on nutritionDay in the Chinese cohort, similar to 22.5% reported in the Poland cohort (42). Our findings highlight prolonged LOS in patients with reduced mobility, consistent with previous reports (15), which is generally associated with unfavorable prognosis. Globally, patients with reduced mobility were more frequently observed to have weight loss (5, 43) and reduced food intake (15, 18). Intriguingly, patients receiving dietary nutrition had a 0.45 lower probability of reduced mobility compared with patients receiving artificial nutrition, and patients who ate completely had a 0.34 lower probability of reduced mobility than those who ate incompletely. Accordingly, the effect of mobility status on discharge home was not obvious in patients both receiving dietary nutrition and eating completely on nutritionDay, who had a lower chance of reduced mobility. We speculate that reduced mobility was possibly prevented by dietary nutrition provided by clinical staff and might be triggered by lower meal consumption. Previous studies have indicated a positive association between dietary patterns and mobility status. The Japanese dietary pattern has been shown to be associated with a lower risk of disability (44, 45). However, actual meal consumption is the premise for modified dietary patterns, and improving dietary quality has been suggested as a potential way to reduce progression of mobility limitations (46). Moreover, the nutritional environment of the ward has been indicated to be associated with patients’ meal consumption and mobility status during hospitalization (15).

To our knowledge, this is the first study to evaluate how inpatients’ mobility status combined with nutritional intervention and meal eaten affect discharge home within 30 days in Chinese hospitalized patients receiving artificial nutrition, dietary nutrition, complete meals, and incomplete meals, and to provide data on the relationship between reduced mobility and nutritional characteristics. We utilized direct data from 2010 to 2020, adjusting for other parameters in the prospective cross-sectional nutritionDay survey to obtain reliable estimates comparable with other patient cohorts. Nevertheless, several potential limitations should be acknowledged. First, the cross-sectional nature of nutritional assessment on a single day limits causal inference regarding the relationship between mobility and nutritional parameters. Second, potential reporting biases exist due to self-reported mobility status from a single-day survey and unbalanced representation across hospitals and departments. Third, detailed levels of mobility status, specific nutritional support, and quantities of meal eaten should be identified in future studies. Fourth, longer follow-up periods beyond discharge home within 30 days need further refinement. Further studies incorporating interventions targeting patients’ physical activity and nutritional care during hospitalization would deepen understanding of the relationship between mobilization, nutritional characteristics, and clinical outcomes.

In conclusion, walking unaided was associated with a higher chance of discharge home, particularly in patients receiving artificial nutrition and those eating incompletely. Patients receiving dietary nutrition had a 0.5 lower probability of reporting reduced mobility compared with those receiving artificial nutrition, and patients who ate completely had a 0.3 lower probability of reduced mobility compared with those eating incompletely. These results enrich scientific evidence on mobility status combined with direct nutritional information during hospitalization to benchmark customized nutritional care. However, this cross-sectional study cannot determine a causal relationship between mobility and nutritional parameters. Further studies are required to validate causal inference and improve inpatients’ mobility. Additionally, future strategies must also account for the long-term effects of mobility patterns on clinical outcomes.

## Data Availability

The datasets presented in this article are not readily available because the raw data supporting the conclusions of this article will be made available by the authors on request. Requests to access the datasets should be directed to zlshe1107@163.com.
