# Supplementary material for: Mobility status, nutritional intervention and meal eaten are associated with discharge home: the nutritionDay study in China
Source: Front Nutr. 2025 Sep 23;12:1631276. doi: 10.3389/fnut.2025.1631276 (PMC12503031; doi:10.3389/fnut.2025.1631276)
Supplement: Supplementary file 1 [file Data_Sheet_1.docx]

Supplementary Material

**Content**

**Supplementary Table S1.** Cox regression analysis of discharged home within 30 days after nutritionDay (n = 5511).

**Supplementary Table S2.** Demographic and nutritional characteristics of patients with mobility status on nutritionDay (n = 5511).

**Supplementary Table S3.** Demographic and nutritional characteristics of patients with mobility status on nutritionDay (Sensitivity analysis without missing value, n=2872).

**Supplementary Table S4.** Demographic and nutritional characteristics of patients with mobility status on nutritionDay (Sensitivity analysis based on digestive disease of primary diagnosis, n=2123).

**Supplementary Table S5.** Demographic and nutritional characteristics of patients with mobility status on nutritionDay (Sensitivity analysis adjusted for pre-hospital functional status, n=5511).

**Supplementary Table S6.** Demographic and nutritional characteristics of patients with mobility status on nutritionDay(Sensitivity analysis adjusted for underlying comorbidities, n=5511).

**Supplementary Figure S1.** Cumulative incidence of discharged home within 30 days after nutritionDay in patients with different nutritional interventions and meal eaten stratified by survey years and regions.

**Supplementary Figure S2.**  Subgroup analysis of associations of mobility, nutritional interventions, and meal eaten with discharged home in patients with dietary nutrition and completely eaten (n=1480).

**Supplementary Figure S3.** Subgroup analysis of associations of mobility, nutritional interventions, and meal eaten with discharged home in patients with artificial nutrition and incompletely eaten (n=1022).

**Supplementary Figure S4.** Subgroup analysis of associations of mobility, nutritional interventions, and meal eaten with discharged home in patients from 2010 to 2015 (n=1590)**.**

**Supplementary Figure S5.** Subgroup analysis of associations of mobility, nutritional interventions, and meal eaten with discharged home in patients from 2016 to 2020 (n=3921)**.**

**Supplementary Figure S6.** Subgroup analysis of associations of mobility, nutritional interventions, and meal eaten with discharged home in patients in eastern region (n=4994)**.**

**Supplementary Figure S7.** Subgroup analysis of associations of mobility, nutritional interventions, and meal eaten with discharged home in patients in western region (n=517).

**Supplementary Figure S8.** Subgroup analysis of associations of mobility, nutritional interventions, and meal eaten with discharged home in patients with artificial nutrition (n=1280).

**Supplementary Figure S9.** Subgroup analysis of associations of mobility, nutritional interventions, and meal eaten with discharged home in patients with dietary nutrition (n=2877).

**Supplementary Figure S10.** Subgroup analysis of associations of mobility, nutritional interventions, and meal eaten with discharged home in patients with completely eaten (n=2087).

**Supplementary Figure S11.** Subgroup analysis of associations of mobility, nutritional interventions, and meal eaten with discharged home in patients with incompletely eaten (n=3252).

**Supplementary Figure S12.** Sensitivity analysis of associations of mobility, nutritional interventions, and meal eaten with discharged home based on digestive disease of primary diagnosis (n=2123).

**Supplementary Figure S13.** Sensitivity analysis of associations of mobility, nutritional interventions, and meal eaten with discharged home with the exclusion of missing values (n=2872).

**Supplementary Figure S14.** Sensitivity analysis of associations of mobility, nutritional interventions, and meal eaten with discharged home adjusted for pre-hospital functional status (n=5511).

**Supplementary Figure S15.** Sensitivity analysis of associations of mobility, nutritional interventions, and meal eaten with discharged home adjusted for underlying comorbidities (n=5511).

**Supplementary Figure S16.** Cumulative incidence of discharged home within 30 days after nutritionDay in patients with different nutritional intervention and meal eaten on the survey days (Sensitivity analysis without missing value and based on digestive disease of primary diagnosis).

**Supplementary Table S1.** Cox regression analysis of discharged home within 30 days after nutritionDay, n = 5511.

| Variable | Category | Univariate analysis | Model Ⅰ | Model Ⅱ | Model Ⅲ |
| --- | --- | --- | --- | --- | --- |
|  |  | HR [95% CI] | HR [95% CI] | HR [95% CI] | HR [95% CI] |
| **Sex** | Female | Reference | Reference | Reference | Reference |
|  | Male | 0.95  [0.89-1.00] | 0.95  [0.89-1.01] | 0.96 [0.89-1.03] | 0.95  [0.89-1.00] |
|  | Missing | 0.86 [0.28-2.67] | 0.74  [0.24-2.33] | 2.40 [0.77-7.55] | 0.56  [0.14-2.26] |
| **Age** | 18-29 years | Reference |  |  |  |
|  | 30-39 years | 0.94 [0.82-1.08] |  |  |  |
|  | 40-49 years | 1.09 [0.96-1.24] |  |  |  |
|  | 50-59 years | 1.08 [0.96-1.22] |  |  |  |
|  | 60-69 years | 1.05  [0.94-1.18] |  |  |  |
|  | 70-79 years | 1.01 [0.89-1.15] |  |  |  |
|  | ≥ 80 years | 0.88 [0.75-1.04] |  |  |  |
| **BMI,** **kg/m^2^** | 18.5-24.9 | Reference | Reference | Reference | Reference |
|  | < 18.5 | 0.77 [0.70-0.84]^***^ | 0.89  [0.81-0.98]^*^ | 0.88  [0.79-0.99]^*^ | 0.88  [0.80-0.97]^*^ |
|  | 25.0-29.9 | 1.13 [1.06-1.21]^***^ | 1.09  [1.02-1.17]^*^ | 1.08  [1.00-1.17] | 1.09  [1.02-1.17]^*^ |
|  | ≥ 30.0 | 1.17 [0.99-1.37] | 1.12  [0.95-1.33] | 1.24  [1.02-1.50]^*^ | 1.14  [0.96-1.35] |
|  | Missing | 1.31 [1.02-1.69]^*^ | 1.22  [0.94-1.58] | 1.00  [0.73-1.38] | 1.18  [0.91-1.53] |
| **Major lesion types** | Cancer | Reference | Reference | Reference | Reference |
|  | Neurological disease | 0.87 [0.77-0.99]^*^ | 1.06  [0.87-1.30] | 1.10  [0.88-1.36] | 1.07  [0.88-1.31] |
|  | Digestive disease | 1.09 [1.01-1.17]^*^ | 1.06  [0.96-1.16] | 1.03  [0.92-1.15] | 1.03  [0.94-1.14] |
|  | Endocrine/  nutritional/  metabolic disease | 0.94  [0.76-1.16] | 0.86  [0.67-1.09] | 0.75  [0.57-1.00]^*^ | 0.81  [0.64-1.04] |
|  | Cardiovascular disease | 0.88 [0.74-1.04] | 1.13  [0.94-1.37] | 1.16  [0.95-1.42] | 1.12  [0.93-1.36] |
|  | Respiratory disease | 1.05 [0.87-1.25] | 1.14  [0.91-1.44] | 1.14  [0.86-1.51] | 1.11  [0.88-1.41] |
|  | Genitourinary disease | 1.18  [1.03-1.36]^*^ | 0.74 [0.57-0.97]^*^ | 0.75  [0.57-1.00]^*^ | 0.72  [0.55-0.95]^*^ |
|  | Orthopaedics diseases | 1.09 [0.92-1.29] | 1.52 [1.22-1.91]^***^ | 1.66 [1.27-2.17]^***^ | 1.48 [1.18-1.86]^***^ |
|  | Other | 0.84 [0.74-0.95]^**^ | 1.03  [0.90-1.19] | 0.99  [0.84-1.17] | 1.02  [0.88-1.18] |
|  | Missing | 1.40 [1.24-1.57]^***^ | 1.70 [1.36-2.13]^***^ | 1.82 [1.41-2.34]^***^ | 1.69  [1.35-2.12]^***^ |
| **Comorbidity** | No | Reference | Reference | Reference | Reference |
|  | Yes | 0.84 [0.79-0.89]^***^ | 0.85 [0.79-0.91]^***^ | 0.82  [0.75-0.90]^***^ | 0.85 [0.79-0.91]^***^ |
|  | Missing | 1.24 [1.11-1.37]^***^ | 1.03  [0.85-1.24] | 0.99  [0.80-1.22] | 1.02  [0.84-1.23] |
| **Previous ICU stay** | No | Reference | Reference | Reference | Reference |
|  | Yes | 0.87 [0.80-0.94]^***^ | 1.01  [0.92-1.12] | 1.02 [0.91-1.14] | 1.03  [0.94-1.14] |
|  | Missing | 0.98 [0.80-1.21] | 0.93  [0.74-1.17] | 1.02 [0.78-1.33] | 0.96  [0.75-1.23] |
| **Mobility** | Mobile | Reference | Reference |  |  |
|  | Reduced mobility | 0.70 [0.66-0.75]^***^ | 0.85  [0.78-0.92]^***^ |  |  |
| **Self-rated health** | Excellent and very good | 0.92 [0.82-1.03] | 0.93  [0.83-1.04] | 0.91 [0.80-1.04] | 0.94  [0.84-1.05] |
|  | Good | Reference | Reference | Reference | Reference |
|  | Fair | 0.81 [0.76-0.87]^***^ | 0.92  [0.85-0.98]^*^ | 0.92 [0.85-1.00] | 0.93 [0.86-0.99]^*^ |
|  | Poor and very poor | 0.72 [0.66-0.80]^***^ | 0.98  [0.88-1.09] | 1.00  [0.89-1.13] | 0.98  [0.88-1.10] |
|  | Missing | 0.72 [0.50-1.03] | 0.79  [0.54-1.16] | 0.75  [0.49-1.15] | 0.78  [0.48-1.27] |
| **Surgical status** | Non-surgical | Reference | Reference | Reference | Reference |
|  | Preoperative | 0.88 [0.82-0.95]^***^ | 0.82 [0.75-0.89]^***^ | 0.79 [0.72-0.88]^***^ | 0.81 [0.74-0.88]^***^ |
|  | Postoperative | 1.20 [1.12-1.28]^***^ | 1.38 [1.27-1.49]^***^ | 1.38 [1.26-1.51]^***^ | 1.36 [1.26-1.48]^***^ |
|  | Undefined or missing | 1.20 [0.74-1.97] | 0.95  [0.57-1.58] | 0.91  [0.51-1.61] | 0.99  [0.59-1.64] |
| **LOS before nutritionDay** | 0-6 days | Reference | Reference | Reference | Reference |
|  | 7-13 days | 0.96 [0.90-1.03] | 1.00  [0.93-1.07] | 1.03  [0.94-1.12] | 1.00  [0.93-1.08] |
|  | 14-20 days | 0.90 [0.81-0.99]^*^ | 0.98  [0.88-1.09] | 1.03 [0.90-1.17] | 0.97  [0.87-1.08] |
|  | ≥ 21 days | 0.51 [0.46-0.57]^***^ | 0.63 [0.56-0.71]^***^ | 0.61 [0.53-0.70]^***^ | 0.63 [0.56-0.71]^***^ |
|  | Missing | 1.03 [0.92-1.15] | 0.79  [0.67-0.94]^**^ | 0.83 [0.68-1.01] | 0.79 [0.66-0.94]^**^ |
| **Number of drugs before admission** | None | Reference | Reference | Reference | Reference |
|  | 1-2 | 0.93 [0.87-1.00]^*^ | 1.01  [0.94-1.09] | 1.02  [0.94-1.12] | 1.01  [0.93-1.09] |
|  | 3-5 | 0.95 [0.88-1.03] | 1.06  [0.97-1.15] | 1.09 [0.98-1.20] | 1.05  [0.96-1.15] |
|  | ＞5 | 0.76 [0.69-0.84]^***^ | 0.99  [0.88-1.11] | 0.98 [0.86-1.12] | 0.99  [0.89-1.11] |
|  | Unknown or missing | 0.77 [0.67-0.87]^***^ | 1.00  [0.87-1.15] | 1.02 [0.87-1.20] | 1.00  [0.87-1.15] |
| **Weight change within last 3 months** | Stable weight | Reference | Reference | Reference | Reference |
|  | Weight loss | 0.81 [0.76-0.86]^***^ | 0.95  [0.88-1.02] | 0.94  [0.87-1.02] | 0.95  [0.89-1.02] |
|  | Weight gain | 1.02 [0.90-1.15] | 1.05  [0.92-1.20] | 1.11 [0.95-1.29] | 1.04  [0.92-1.19] |
|  | Unsure or missing | 0.82 [0.74-0.91]^***^ | 0.88  [0.79-0.98]^*^ | 0.88  [0.78-0.99]^*^ | 0.88  [0.79-0.98]^*^ |
| **Food intake in the previous week** | More than normal or normal | Reference | Reference | Reference | Reference |
|  | A little less than normal | 0.87 [0.81-0.97]^**^ | 0.94  [0.86-1.03] | 0.92  [0.82-1.02] | 0.95  [0.87-1.04] |
|  | Less than half of normal | 0.74 [0.69-0.80]^***^ | 0.86  [0.79-0.94]^**^ | 0.88 [0.79-0.97]^*^ | 0.87 [0.80-0.95]^**^ |
|  | Missing | 1.03 [0.74-1.42] | 1.11  [0.79-1.56] | 1.08  [0.74-1.58] | 1.04  [0.70-1.56] |
| **Eating on nutritionDay** | Eaten all | Reference | Reference | Reference |  |
|  | Eaten half | 1.04 [0.96-1.12] | 1.08  [1.00-1.17] | 1.10  [1.00-1.20] |  |
|  | Eaten quarter | 0.91 [0.82-1.01] | 1.03  [0.92-1.15] | 1.01  [0.89-1.16] |  |
|  | Eaten nothing | 0.80 [0.75-0.86]^***^ | 0.93  [0.85-1.01] | 0.86 [0.78-0.96]^**^ |  |
|  | Missing | 0.80 [0.68-0.94]^**^ | 1.18  [0.98-1.41] | 1.17  [0.94-1.46] |  |
| **Nutritional intervention** | Artificial nutrition | Reference | Reference |  | Reference |
|  | Dietary nutrition | 1.65 [1.54-1.78]^***^ | 1.57 [1.43-1.73]^***^ |  | 1.66 [1.52-1.82]^***^ |
|  | Multi-form of artificial and dietary nutrition | 1.50 [1.35-1.67]^***^ | 1.37 [1.22-1.55]^***^ |  | 1.44 [1.28-1.62]^***^ |
|  | None | 1.67 [1.41-1.98]^***^ | 1.39 [1.16-1.66]^***^ |  | 1.42 [1.19-1.70]^***^ |
|  | Unsure or missing | 1.54 [1.38-1.71]^***^ | 1.55 [1.38-1.74]^***^ |  | 1.59  [1.41-1.80]^***^ |
| **Nutritional intervention with mobility status** | Artificial nutrition with reduced mobility |  |  | Reference |  |
|  | Artificial nutrition with mobile |  |  | 1.17 [1.02-1.34]^*^ |  |
|  | Dietary nutrition with reduced mobility |  |  | 1.63 [1.39-1.91]^***^ |  |
|  | Dietary nutrition with mobile |  |  | 1.80 [1.57-2.08]^***^ |  |
| **Meal eaten with mobility status** | Incompletely eaten with reduced mobility |  |  |  | Reference |
|  | Incompletely eaten with mobile |  |  |  | 1.25 [1.14-1.37]^***^ |
|  | Eaten all with  reduced mobility |  |  |  | 1.15  [0.98-1.36] |
|  | Eaten all with  mobile |  |  |  | 1.19 [1.08-1.32]^***^ |

Cox regression models with covariates of departments, survey year, hospital location, sex, BMI, weight change within the last 3 months, major lesion types, comorbidity, food intake in the previous week, previous ICU stay, self-rated health, surgical status, LOS before nutritionDay, and number of drugs before admission. Model I: Multivariable analysis with nutritional intervention, meal eaten and mobility status. Model II: Meal eaten and combined nutritional intervention with mobility status added to the multivariable analysis. Model III: Nutritional intervention and combined meal eaten with mobility status added to the multivariable analysis. All data are presented as HR and 95% CI. * *p* < 0.05, ** *p* < 0.01, *** *p* < 0.001. HR, hazard ratio; CI, confidence interval; BMI, body mass index; ICU, intensive care unit; LOS, length of hospital stay.

**Supplementary Table S2.** Demographic and nutritional characteristics of patients with mobility status on nutritionDay, n=5511

| Variable | All  n (%) | Univariate analysis | | | | Multivariable analysis |
| --- | --- | --- | --- | --- | --- | --- |
|  |  | Mobile  n (%) | Reduced mobility  n (%) | *p* value | OR [95% CI] | OR [95% CI] |
|  | 5511 | 4232 | 1279 |  |  |  |
| Sex |  |  |  | 0.814 |  |  |
| Female | 2231 (40.5%) | 1704 (40.3%) | 527 (41.2%) |  | Reference |  |
| Male | 3276 (59.4%) | 2525 (59.7%) | 751 (58.7%) |  | 0.96 [0.85-1.09] |  |
| Missing | 4 (0.1%) | 3 (0.1%) | 1 (0.1%) |  | 1.08 [0.11-10.38] |  |
| Age |  |  |  | < 0.001 |  |  |
| 18-29 years | 422 (7.7%) | 333 (7.9%) | 89 (7.0%) |  | Reference | Reference |
| 30-39 years | 553 (10.0%) | 445 (10.5%) | 108 (8.4%) |  | 0.91 [0.66-1.24] | 1.16 [0.80-1.69] |
| 40-49 years | 803 (14.6%) | 639 (15.1%) | 164 (12.8%) |  | 0.96 [0.72-1.28] | 1.17 [0.82-1.66] |
| 50-59 years | 1203 (21.8%) | 986 (23.3%) | 217 (17.0%) |  | 0.82 [0.62-1.09] | 1.13 [0.80-1.58] |
| 60-69 years | 1416 (25.7%) | 1090 (25.8%) | 326 (25.5%) |  | 1.12 [0.86-1.46] | 1.53 [1.10-2.14] ^*^ |
| 70-79 years | 814 (14.8%) | 582 (13.8%) | 232 (18.1%) |  | 1.49 [1.13-1.97] ^**^ | 1.98 [1.39-2.82] ^***^ |
| ≥ 80 years | 300 (5.4%) | 157 (3.7%) | 143 (11.2%) |  | 3.41 [2.46-4.72] ^***^ | 4.63 [3.07-7.00] ^***^ |
| BMI, kg/m² |  |  |  | < 0.001 |  |  |
| 18.5-24.9 | 3291 (59.7%) | 2522 (59.6%) | 769 (60.1%) |  | Reference | Reference |
| < 18.5 | 671 (12.2%) | 477 (11.3%) | 194 (15.2%) |  | 1.33 [1.11-1.61] ^**^ | 1.02 [0.81-1.29] |
| 25.0-29.9 | 1305 (23.7%) | 1038 (24.5%) | 267 (20.9%) |  | 0.84 [0.72-0.99] ^*^ | 1.02 [0.85-1.23] |
| ≥ 30.0 | 175 (3.2%) | 146 (3.4%) | 29 (2.3%) |  | 0.65 [0.43-0.98] ^*^ | 0.86 [0.54-1.37] |
| Missing | 69 (1.3%) | 49 (1.2%) | 20 (1.6%) |  | 1.34 [0.79-2.27] | 1.98 [1.05-3.74] ^*^ |
| Major lesion types |  |  |  | < 0.001 |  |  |
| Cancer | 1351 (24.5%) | 1042 (24.6%) | 309 (24.2%) |  | Reference | Reference |
| Neurological disease | 372 (6.8%) | 227 (5.4%) | 145 (11.3%) |  | 2.15 [1.69-2.75] ^***^ | 1.08 [0.68-1.73] |
| Digestive disease | 2123 (38.5%) | 1755 (41.5%) | 368 (28.8%) |  | 0.71 [0.60-0.84] ^***^ | 0.66 [0.52-0.83] ^***^ |
| Endocrine/  nutritional/  metabolic disease | 110 (2.0%) | 103 (2.4%) | 7 (0.5%) |  | 0.23 [0.11-0.50] ^***^ | 0.29 [0.12-0.70] ^**^ |
| Cardiovascular disease | 182 (3.3%) | 123 (2.9%) | 59 (4.6%) |  | 1.62 [1.16-2.26] ^**^ | 1.38 [0.87-2.18] |
| Respiratory disease | 149 (2.7%) | 105 (2.5%) | 44 (3.4%) |  | 1.41 [0.97-2.05] | 1.11 [0.64-1.93] |
| Genitourinary disease | 267 (4.8%) | 225 (5.3%) | 42 (3.3%) |  | 0.63 [0.44-0.90] ^*^ | 0.82 [0.39-1.68] |
| Orthopaedics diseases | 160 (2.9%) | 87 (2.1%) | 73 (5.7%) |  | 2.83 [2.02-3.96] ^***^ | 1.22 [0.72-2.08] |
| Other | 365 (6.6%) | 240 (5.7%) | 125 (9.8%) |  | 1.76 [1.37-2.26] ^***^ | 1.00 [0.71-1.42] |
| Missing | 432 (7.8%) | 325 (7.7%) | 107 (8.4%) |  | 1.11 [0.86-1.43] | 1.06 [0.59-1.91] |
| Comorbidity |  |  |  | < 0.001 |  |  |
| No | 1785 (32.4%) | 1474 (34.8%) | 311 (24.3%) |  |  | Reference |
| Yes | 3218 (58.4%) | 2366 (55.9%) | 852 (66.6%) |  | 1.71 [1.48-1.97] ^***^ | 1.22 [1.00-1.48] ^*^ |
| Missing | 508 (9.2%) | 392 (9.3%) | 116 (9.1%) |  | 1.40 [1.10-1.78] ^**^ | 1.15 [0.69-1.92] |
| Previous ICU stay |  |  |  | < 0.001 |  |  |
| No | 4642 (84.2%) | 3718 (87.9%) | 924 (72.2%) |  | Reference | Reference |
| Yes | 760 (13.8%) | 435 (10.3%) | 325 (25.4%) |  | 3.01 [2.56-3.53] ^***^ | 1.60 [1.29-1.97] ^***^ |
| Missing | 109 (2.0%) | 79 (1.9%) | 30 (2.3%) |  | 1.53 [1.00-2.34] | 0.76 [0.43-1.35] |
| End-stage disease |  |  |  | < 0.001 |  |  |
| No | 4730 (85.8%) | 3652 (86.3%) | 1078 (84.3%) |  | Reference |  |
| Yes | 252 (4.6%) | 165 (3.9%) | 87 (6.8%) |  | 1.79 [1.37-2.34] ^***^ | 1.19 [0.85-1.65] |
| Unknown or missing | 529 (9.6%) | 415 (9.8%) | 114 (8.9%) |  | 0.93 [0.75-1.16] | 0.95 [0.71-1.25] |
| Number of drugs before admission |  |  |  | < 0.001 |  |  |
| None | 1663 (30.2%) | 1385 (32.7%) | 278 (21.7%) |  | Reference | Reference |
| 1-2 | 1743 (31.6%) | 1389 (32.8%) | 354 (27.7%) |  | 1.27 [1.07-1.51] ^**^ | 1.09 [0.89-1.34] |
| 3-5 | 1136 (20.6%) | 837 (19.8%) | 299 (23.4%) |  | 1.78 [1.48-2.14] ^***^ | 1.22 [0.97-1.53] |
| ＞5 | 629 (11.4%) | 394 (9.3%) | 235 (18.4%) |  | 2.97 [2.42-3.65] ^***^ | 1.72 [1.31-2.26] ^***^ |
| Unknown or missing | 340 (6.2%) | 227 (5.4%) | 113 (8.8%) |  | 2.48 [1.91-3.22] ^***^ | 1.75 [1.27-2.40] ^***^ |
| Surgical status |  |  |  | < 0.001 |  |  |
| Non-surgical | 2574 (46.7%) | 2052 (48.5%) | 522 (40.8%) |  | Reference | Reference |
| Preoperative | 1208 (21.9%) | 1018 (24.1%) | 190 (14.9%) |  | 0.73 [0.61-0.88] ^***^ | 0.85 [0.68-1.08] |
| Postoperative | 1702 (30.9%) | 1143 (27.0%) | 559 (43.7%) |  | 1.92 [1.67-2.21] ^***^ | 1.72 [1.40-2.10] ^***^ |
| Undefined | 27 (0.5%) | 19 (0.4%) | 8 (0.6%) |  | 1.66 [0.72-3.80] | 1.96 [0.67-5.74] |
| Self-rated health |  |  |  | < 0.001 |  |  |
| Excellent and very good | 433 (7.9%) | 385 (9.1%) | 48 (3.8%) |  | 0.69 [0.50-0.96] ^*^ | 0.77 [0.53-1.12] |
| Good | 1754 (31.8%) | 1487 (35.1%) | 267 (20.9%) |  | Reference | Reference |
| Fair | 2603 (47.2%) | 1942 (45.9%) | 661 (51.7%) |  | 1.90 [1.62-2.22] ^***^ | 1.66 [1.38-2.00] ^***^ |
| Poor and very poor | 689 (12.5%) | 393 (9.3%) | 296 (23.1%) |  | 4.19 [3.44-5.12] ^***^ | 3.16 [2.47-4.05] ^***^ |
| Missing | 32 (0.6%) | 25 (0.6%) | 7 (0.5%) |  | 1.56 [0.67-3.64] | 1.47 [0.52-4.10] |
| Weight change within last 3 months |  |  |  | < 0.001 |  |  |
| Stable weight | 2501 (45.4%) | 2051 (48.5%) | 450 (35.2%) |  | Reference | Reference |
| Weight loss | 2156 (39.1%) | 1541 (36.4%) | 615 (48.1%) |  | 1.82 [1.58-2.09] ^***^ | 1.14 [0.95-1.36] |
| Weight gain | 320 (5.8%) | 269 (6.4%) | 51 (4.0%) |  | 0.86 [0.63-1.19] | 0.92 [0.64-1.32] |
| Unsure or missing | 534 (9.7%) | 371 (8.8%) | 163 (12.7%) |  | 2.00 [1.62-2.47] ^***^ | 1.53 [1.18-1.98] ^**^ |
| LOS before nutritionDay |  |  |  | < 0.001 |  |  |
| 0-6 days | 2654 (48.2%) | 2246 (53.1%) | 408 (31.9%) |  | Reference | Reference |
| 7-13 days | 1342 (24.4%) | 944 (22.3%) | 398 (31.1%) |  | 2.32 [1.98-2.72] ^***^ | 1.51 [1.25-1.83] ^***^ |
| 14-20 days | 521 (9.4%) | 351 (8.3%) | 170 (13.3%) |  | 2.67 [2.16-3.29] ^***^ | 1.31 [1.01-1.69] ^*^ |
| ≥ 21 days | 542 (9.8%) | 332 (7.8%) | 210 (16.4%) |  | 3.48 [2.84-4.26] ^***^ | 1.36 [1.06-1.76] ^*^ |
| Missing | 452 (8.2%) | 359 (8.5%) | 93 (7.3%) |  | 1.43 [1.11-1.83] ^**^ | 1.38 [0.91-2.10] |
| Food intake in the previous week |  |  |  | < 0.001 |  |  |
| More than normal or normal | 3661 (66.4%) | 3002 (70.9%) | 659 (51.5%) |  | Reference | Reference |
| A little less than normal | 707 (12.8%) | 492 (11.6%) | 215 (16.8%) |  | 1.99 [1.66-2.39] ^***^ | 1.08 [0.86-1.36] |
| Less than half of normal | 1104 (20.0%) | 706 (16.7%) | 398 (31.1%) |  | 2.57 [2.21-2.98] ^***^ | 1.34 [1.10-1.64] ^**^ |
| Missing | 39 (0.7%) | 32 (0.8%) | 7 (0.5%) |  | 1.00 [0.44-2.27] | 0.61 [0.25-1.53] |
| Nutritional intervention |  |  |  | < 0.001 |  |  |
| Artificial nutrition | 1280 (23.2%) | 813 (19.2%) | 467 (36.5%) |  | Reference | Reference |
| Dietary nutrition | 2877 (52.2%) | 2393 (56.5%) | 484 (37.8%) |  | 0.35 [0.30-0.41] ^***^ | 0.45 [0.36-0.55] ^***^ |
| Multi-form of artificial and dietary nutrition | 569 (10.3%) | 408 (9.6%) | 161 (12.6%) |  | 0.69 [0.55-0.85] ^***^ | 0.70 [0.54-0.92] ^**^ |
| None | 175 (3.2%) | 137 (3.2%) | 38 (3.0%) |  | 0.48 [0.33-0.70] ^***^ | 0.90 [0.58-1.39] |
| Unsure or missing | 610 (11.1%) | 481 (11.4%) | 129 (10.1%) |  | 0.47 [0.37-0.58] ^***^ | 0.64 [0.48-0.84] ^**^ |
| Eating on nutritionDay |  |  |  | < 0.001 |  |  |
| Incompletely eaten | 3252 (59.0%) | 2285 (54.0%) | 967 (75.6%) |  | Reference | Reference |
| Completely eaten | 2087 (37.9%) | 1832 (43.3%) | 255 (19.9%) |  | 0.33 [0.28-0.38] ^***^ | 0.34 [0.28-0.41] ^***^ |
| Missing | 172 (3.1%) | 115 (2.7%) | 57 (4.5%) |  | 1.17 [0.85-1.62] | 0.91 [0.61-1.37] |

Comparisons of patients groups between mobile patients and reduced mobility patients were performed by the Chi-square test, Fisher's exact test or Wilcoxon rank-sum test, as appropriate. *p* value, mobile patients vs. reduced mobility patients. Significant variables with *p* < 0.05 in univariate analysis were included in a logistic regression analysis to assess the association between reduced mobility and patients’ characteristics in multivariable analysis. BMI, body mass index. OR, odds ratio; CI, confidence interval; ICU, intensive care unit; LOS, length of hospital stay. * *p* < 0.05, ** *p* < 0.01, *** *p* < 0.001.

**Supplementary Table S3.** Demographic and nutritional characteristics of patients with mobility status on nutritionDay, n=2872 (Sensitivity analysis without missing values).

| Variable | All  n (%) | Univariate analysis | | | | Multivariable analysis |
| --- | --- | --- | --- | --- | --- | --- |
|  |  | Mobile  n (%) | Reduced mobility  n (%) | *P* value | OR [95% CI] | OR [95% CI] |
|  | 2872 | 2255 | 617 |  |  |  |
| Sex |  |  |  | 0.815 |  |  |
| Female | 1145 (39.9%) | 896 (39.7%) | 249 (40.4%) |  | Reference |  |
| Male | 1727 (60.1%) | 1359 (60.3%) | 368 (59.6%) |  | 0.97 [0.81-1.17] |  |
| Age |  |  |  | < 0.001 |  |  |
| 18-29 years | 227 (7.9%) | 194 (8.6%) | 33 (5.3%) |  | Reference | Reference |
| 30-39 years | 292 (10.2%) | 239 (10.6%) | 53 (8.6%) |  | 1.30 [0.81-2.09] | 1.59 [0.90-2.80] |
| 40-49 years | 410 (14.3%) | 333 (14.8%) | 77 (12.5%) |  | 1.36 [0.87-2.12] | 1.32 [0.77-2.28] |
| 50-59 years | 661 (23.0%) | 556 (24.7%) | 105 (17.0%) |  | 1.11 [0.73-1.70] | 1.24 [0.73-2.10] |
| 60-69 years | 719 (25.0%) | 554 (24.6%) | 165 (26.7%) |  | 1.75 [1.16-2.63] ^**^ | 2.12 [1.26-3.55] ^**^ |
| 70-79 years | 405 (14.1%) | 305 (13.5%) | 100 (16.2%) |  | 1.93 [1.25-2.97] ^**^ | 2.30 [1.33-3.98] ^**^ |
| ≥ 80 years | 158 (5.5%) | 74 (3.3%) | 84 (13.6%) |  | 6.67 [4.11-10.82] ^***^ | 9.19 [4.89-17.30] ^***^ |
| BMI, kg/m² |  |  |  | < 0.01 |  |  |
| 18.5-24.9 | 1706 (59.4%) | 1330 (59.0%) | 376 (60.9%) |  | Reference | Reference |
| < 18.5 | 354 (12.3%) | 270 (12.0%) | 84 (13.6%) |  | 1.10 [0.84-1.44] | 0.81 [0.58-1.15] |
| 25.0-29.9 | 714 (24.9%) | 572 (25.4%) | 142 (23.0%) |  | 0.88 [0.71-1.09] | 1.04 [0.79-1.36] |
| ≥ 30.0 | 98 (3.4%) | 83 (3.7%) | 15 (2.4%) |  | 0.64 [0.36-1.12] | 0.93 [0.48-1.81] |
| Major lesion types |  |  |  | < 0.001 |  |  |
| Cancer | 651 (22.7%) | 500 (22.2%) | 151 (24.5%) |  | Reference | Reference |
| Neurological disease | 224 (7.8%) | 152 (6.7%) | 72 (11.7%) |  | 1.57 [1.12-2.19] ^**^ | 0.45 [0.22-0.91] ^*^ |
| Digestive disease | 1190 (41.4%) | 1003 (44.5%) | 187 (30.3%) |  | 0.62 [0.49-0.78] ^***^ | 0.55 [0.39-0.79] ^***^ |
| Endocrine/  nutritional/  metabolic disease | 72 (2.5%) | 68 (3.0%) | 4 (0.6%) |  | 0.19 [0.07-0.54] ^**^ | 0.27 [0.08-0.90] ^*^ |
| Cardiovascular disease | 142 (4.9%) | 95 (4.2%) | 47 (7.6%) |  | 1.64 [1.10-2.43] ^*^ | 1.19 [0.66-2.13] |
| Respiratory disease | 105 (3.7%) | 82 (3.6%) | 23 (3.7%) |  | 0.93 [0.57-1.53] | 0.56 [0.25-1.26] |
| Genitourinary disease | 208 (7.2%) | 178 (7.9%) | 30 (4.9%) |  | 0.56 [0.36-0.86] ^**^ | 0.35 [0.07-1.70] |
| Orthopaedics diseases | 84 (2.9%) | 50 (2.2%) | 34 (5.5%) |  | 2.25 [1.40-3.61] ^***^ | 0.83 [0.38-1.78] |
| Other | 196 (6.8%) | 127 (5.6%) | 69 (11.2%) |  | 1.80 [1.27-2.54] ^***^ | 0.89 [0.53-1.49] |
| Comorbidity |  |  |  | < 0.001 |  |  |
| No | 1041 (36.2%) | 879 (39.0%) | 162 (26.3%) |  |  | Reference |
| Yes | 1831 (63.8%) | 1376 (61.0%) | 455 (73.7%) |  | 1.79 [1.47-2.19] ^***^ | 1.19 [0.90-1.56] |
| Previous ICU stay |  |  |  | < 0.001 |  |  |
| No | 2529 (88.1%) | 2060 (91.4%) | 469 (76.0%) |  | Reference | Reference |
| Yes | 343 (11.9%) | 195 (8.6%) | 148 (24.0%) |  | 3.33 [2.63-4.22] ^***^ | 2.04 [1.48-2.81] ^***^ |
| End-stage disease |  |  |  | < 0.01 |  |  |
| No | 2735 (95.2%) | 2162 (95.9%) | 573 (92.9%) |  | Reference |  |
| Yes | 137 (4.8%) | 93 (4.1%) | 44 (7.1%) |  | 1.79 [1.23-2.59] ^**^ | 1.31 [0.82-2.08] |
| Number of drugs before admission |  |  |  | < 0.001 |  |  |
| None | 920 (32.0%) | 776 (34.4%) | 144 (23.3%) |  | Reference | Reference |
| 1-2 | 975 (33.9%) | 785 (34.8%) | 190 (30.8%) |  | 1.30 [1.03-1.66] ^*^ | 1.10 [0.82-1.47] |
| 3-5 | 579 (20.2%) | 450 (20.0%) | 129 (20.9%) |  | 1.54 [1.19-2.01] ^**^ | 0.96 [0.68-1.34] |
| ＞5 | 398 (13.9%) | 244 (10.8%) | 154 (25.0%) |  | 3.40 [2.60-4.45] ^***^ | 1.90 [1.29-2.80] ^***^ |
| Surgical status |  |  |  | < 0.001 |  |  |
| Non-surgical | 1259 (43.8%) | 1030 (45.7%) | 229 (37.1%) |  | Reference | Reference |
| Preoperative | 627 (21.8%) | 534 (23.7%) | 93 (15.1%) |  | 0.78 [0.60-1.02] | 0.99 [0.69-1.43] |
| Postoperative | 986 (34.3%) | 691 (30.6%) | 295 (47.8%) |  | 1.92 [1.58-2.34] ^***^ | 2.09 [1.53-2.85] ^***^ |
| Self-rated health |  |  |  | < 0.001 |  |  |
| Excellent and very good | 205 (7.1%) | 185 (8.2%) | 20 (3.2%) |  | 0.71 [0.43-1.17] | 0.78 [0.44-1.38] |
| Good | 926 (32.2%) | 804 (35.7%) | 122 (19.8%) |  | Reference | Reference |
| Fair | 1395 (48.6%) | 1071 (47.5%) | 324 (52.5%) |  | 1.99 [1.59-2.50] ^***^ | 1.69 [1.28-2.23] ^***^ |
| Poor and very poor | 346 (12.0%) | 195 (8.6%) | 151 (24.5%) |  | 5.10 [3.84-6.79] ^***^ | 3.97 [2.74-5.75] ^***^ |
| Weight change within last 3 months |  |  |  | < 0.001 |  |  |
| Stable weight | 1554 (54.1%) | 1282 (56.9%) | 272 (44.1%) |  | Reference | Reference |
| Weight loss | 1166 (40.6%) | 850 (37.7%) | 316 (51.2%) |  | 1.75 [1.46-2.11] ^***^ | 1.06 [0.83-1.35] |
| Weight gain | 152 (5.3%) | 123 (5.5%) | 29 (4.7%) |  | 1.11 [0.73-1.70] | 1.44 [0.86-2.41] |
| LOS before nutritionDay |  |  |  | < 0.001 |  |  |
| 0-6 days | 1482 (51.6%) | 1273 (56.5%) | 209 (33.9%) |  | Reference | Reference |
| 7-13 days | 780 (27.2%) | 569 (25.2%) | 211 (34.2%) |  | 2.26 [1.82-2.80] ^***^ | 1.30 [0.99-1.70] |
| 14-20 days | 296 (10.3%) | 204 (9.0%) | 92 (14.9%) |  | 2.75 [2.06-3.66] ^***^ | 1.26 [0.88-1.81] |
| ≥ 21 days | 314 (10.9%) | 209 (9.3%) | 105 (17.0%) |  | 3.06 [2.32-4.03] ^***^ | 1.08 [0.75-1.54] |
| Food intake in the previous week |  |  |  | < 0.001 |  |  |
| More than normal or normal | 1953 (68.0%) | 1627 (72.2%) | 326 (52.8%) |  | Reference | Reference |
| A little less than normal | 372 (13.0%) | 264 (11.7%) | 108 (17.5%) |  | 2.04 [1.58-2.63] ^***^ | 0.89 [0.64-1.25] |
| Less than half of normal | 547 (19.0%) | 364 (16.1%) | 183 (29.7%) |  | 2.51 [2.03-3.11] ^***^ | 1.23 [0.91-1.66] |
| Nutritional intervention |  |  |  | < 0.001 |  |  |
| Artificial nutrition | 696 (24.2%) | 459 (20.4%) | 237 (38.4%) |  | Reference | Reference |
| Dietary nutrition | 1721 (59.9%) | 1469 (65.1%) | 252 (40.8%) |  | 0.33 [0.27-0.41] ^***^ | 0.41 [0.31-0.55] ^***^ |
| Multi-form of artificial and dietary nutrition | 364 (12.7%) | 252 (11.2%) | 112 (18.2%) |  | 0.86 [0.66-1.13] | 0.78 [0.55-1.11] |
| None | 91 (3.2%) | 75 (3.3%) | 16 (2.6%) |  | 0.41 [0.24-0.72] ^**^ | 0.91 [0.47-1.75] |
| Eating on nutritionDay |  |  |  | < 0.001 |  |  |
| Incompletely eaten | 1632 (56.8%) | 1157 (51.3%) | 475 (77.0%) |  | Reference | Reference |
| Completely eaten | 1240 (43.2%) | 1098 (48.7%) | 142 (23.0%) |  | 0.32 [0.26-0.39] ^***^ | 0.31 [0.23-0.40] ^***^ |

Comparisons of patients groups between mobile patients and reduced mobility patients were performed by the Chi-square test, Fisher's exact test or Wilcoxon rank-sum test, as appropriate. *p* value, mobile patients vs. reduced mobility patients. Significant variables with *p* < 0.05 in univariate analysis were included in a logistic regression analysis to assess the association between reduced mobility and patients’ characteristics in multivariable analysis. BMI, body mass index. OR, odds ratio; CI, confidence interval; ICU, intensive care unit; LOS, length of hospital stay. * *p* < 0.05, ** *p* < 0.01, *** *p* < 0.001.

**Supplementary Table S4.** Demographic and nutritional characteristics of patients with mobility status on nutritionDay, n=2123 (Sensitivity analysis based on digestive disease of primary diagnosis).

| Variable | All  n (%) | Univariate analysis | | | | Multivariable analysis |
| --- | --- | --- | --- | --- | --- | --- |
|  |  | Mobile  n (%) | Reduced mobility  n (%) | *P* value | OR [95% CI] | OR [95% CI] |
|  | 2123 | 1755 | 368 |  |  |  |
| Sex |  |  |  | 0.303 |  |  |
| Female | 898 (42.3%) | 738 (42.1%) | 160 (43.5%) |  | Reference |  |
| Male | 1223 (57.6%) | 1016 (57.9%) | 207 (56.3%) |  | 0.94 [0.75-1.18] |  |
| Missing | 2 (0.1%) | 1 (0.1%) | 1 (0.3%) |  | 4.61 [0.29-74.13] |  |
| Age |  |  |  | < 0.001 |  |  |
| 18-29 years | 199 (9.4%) | 176 (10.0%) | 23 (6.3%) |  | Reference | Reference |
| 30-39 years | 257 (12.1%) | 216 (12.3%) | 41 (11.1%) |  | 1.45 [0.84-2.51] | 1.69 [0.91-3.14] |
| 40-49 years | 325 (15.3%) | 278 (15.8%) | 47 (12.8%) |  | 1.29 [0.76-2.21] | 1.45 [0.79-2.68] |
| 50-59 years | 472 (22.2%) | 419 (23.9%) | 53 (14.4%) |  | 0.97 [0.58-1.63] | 1.10 [0.60-1.99] |
| 60-69 years | 508 (23.9%) | 414 (23.6%) | 94 (25.5%) |  | 1.74 [1.07-2.83] ^*^ | 2.24 [1.26-4.00] ^**^ |
| 70-79 years | 258 (12.2%) | 189 (10.8%) | 69 (18.8%) |  | 2.79 [1.67-4.67] ^***^ | 3.19 [1.72-5.92] ^***^ |
| ≥ 80 years | 104 (4.9%) | 63 (3.6%) | 41 (11.1%) |  | 4.98 [2.77-8.95] ^***^ | 7.48 [3.68-15.23] ^***^ |
| BMI, kg/m² |  |  |  | < 0.01 |  |  |
| 18.5-24.9 | 1204 (56.7%) | 994 (56.6%) | 210 (57.1%) |  | Reference | Reference |
| < 18.5 | 349 (16.4%) | 272 (15.5%) | 77 (20.9%) |  | 1.34 [1.00-1.80] | 0.97 [0.68-1.40] |
| 25.0-29.9 | 471 (22.2%) | 402 (22.9%) | 69 (18.8%) |  | 0.81 [0.60-1.09] | 1.05 [0.75-1.48] |
| ≥ 30.0 | 52 (2.4%) | 49 (2.8%) | 3 (0.8%) |  | 0.29 [0.09-0.94] ^*^ | 0.33 [0.09-1.27] |
| Missing | 47 (2.2%) | 38 (2.2%) | 9 (2.4%) |  | 1.12 [0.53-2.35] | 2.29 [0.93-5.64] |
| Comorbidity |  |  |  | < 0.001 |  |  |
| No | 1058 (49.8%) | 924 (52.6%) | 134 (36.4%) |  |  | Reference |
| Yes | 1006 (47.4%) | 782 (44.6%) | 224 (60.9%) |  | 1.98 [1.56-2.50] ^***^ | 1.54 [1.14-2.09] ^**^ |
| Missing | 59 (2.8%) | 49 (2.8%) | 10 (2.7%) |  | 1.41 [0.70-2.84] | 1.46 [0.62-3.45] |
| Previous ICU stay |  |  |  | < 0.001 |  |  |
| No | 1879 (88.5%) | 1601 (91.2%) | 278 (75.5%) |  | Reference | Reference |
| Yes | 219 (10.3%) | 136 (7.7%) | 83 (22.6%) |  | 3.51 [2.60-4.75] ^***^ | 1.98 [1.36-2.87] ^***^ |
| Missing | 25 (1.2%) | 18 (1.0%) | 7 (1.9%) |  | 2.24 [0.93-5.41] | 2.31 [0.79-6.75] |
| End-stage disease |  |  |  | < 0.001 |  |  |
| No | 1979 (93.2%) | 1653 (94.2%) | 326 (88.6%) |  | Reference |  |
| Yes | 53 (2.5%) | 34 (1.9%) | 19 (5.2%) |  | 2.83 [1.60-5.03] ^***^ | 1.55 [0.77-3.11] |
| Unknown or missing | 91 (4.3%) | 68 (3.9%) | 23 (6.3%) |  | 1.72 [1.05-2.79] ^*^ | 1.15 [0.64-2.05] |
| Number of drugs before admission |  |  |  | < 0.001 |  |  |
| None | 762 (35.9%) | 666 (37.9%) | 96 (26.1%) |  | Reference | Reference |
| 1-2 | 697 (32.8%) | 580 (33.0%) | 117 (31.8%) |  | 1.40 [1.05-1.87] ^*^ | 1.02 [0.73-1.43] |
| 3-5 | 357 (16.8%) | 292 (16.6%) | 65 (17.7%) |  | 1.54 [1.10-2.18] ^*^ | 0.99 [0.66-1.49] |
| ＞5 | 184 (8.7%) | 124 (7.1%) | 60 (16.3%) |  | 3.36 [2.31-4.89] ^***^ | 1.68 [1.04-2.70] ^*^ |
| Unknown or missing | 123 (5.8%) | 93 (5.3%) | 30 (8.2%) |  | 2.24 [1.41-3.56] ^***^ | 1.33 [0.75-2.34] |
| Surgical status |  |  |  | < 0.001 |  |  |
| Non-surgical | 963 (45.4%) | 831 (48.5%) | 132 (40.8%) |  | Reference | Reference |
| Preoperative | 449 (21.1%) | 397 (24.1%) | 52 (14.9%) |  | 0.82 [0.59-1.16] | 1.04 [0.69-1.55] |
| Postoperative | 706 (33.3%) | 523 (27.0%) | 183 (43.7%) |  | 2.20 [1.72-2.83] ^***^ | 2.59 [1.86-3.61] ^***^ |
| Undefined | 5 (0.2%) | 4 (0.4%) | 1 (0.6%) |  | 1.57 [0.17-14.19] | 0.91 [0.08-10.69] |
| Self-rated health |  |  |  | < 0.01 |  |  |
| Excellent and very good | 160 (7.5%) | 144 (8.2%) | 16 (4.3%) |  | 1.02 [0.57-1.81] | 1.30 [0.69-2.46] |
| Good | 672 (31.6%) | 606 (34.5%) | 66 (17.9%) |  | Reference | Reference |
| Fair | 1022 (48.1%) | 837 (47.7%) | 185 (50.3%) |  | 2.03 [1.50-2.74] ^***^ | 1.89 [1.34-2.67] ^***^ |
| Poor and very poor | 257 (12.1%) | 157 (8.9%) | 100 (27.2%) |  | 5.85 [4.09-8.36] ^***^ | 4.18 [2.69-6.49] ^***^ |
| Missing | 12 (0.6%) | 11 (0.6%) | 1 (0.3%) |  | 0.83 [0.11-6.56] | 0.32 [0.03-3.19] |
| Weight change within last 3 months |  |  |  | < 0.001 |  |  |
| Stable weight | 992 (46.7%) | 866 (49.3%) | 126 (34.2%) |  | Reference | Reference |
| Weight loss | 860 (40.5%) | 674 (38.4%) | 186 (50.5%) |  | 1.90 [1.48-2.43] ^***^ | 1.04 [0.76-1.41] |
| Weight gain | 114 (5.4%) | 97 (5.5%) | 17 (4.6%) |  | 1.20 [0.70-2.08] | 1.44 [0.76-2.71] |
| Unsure or missing | 157 (7.4%) | 118 (6.7%) | 39 (10.6%) |  | 2.27 [1.51-3.41] ^***^ | 1.64 [1.02-2.65] ^*^ |
| LOS before nutritionDay |  |  |  | < 0.001 |  |  |
| 0-6 days | 999 (47.1%) | 868 (49.5%) | 131 (35.6%) |  | Reference | Reference |
| 7-13 days | 500 (23.6%) | 397 (22.6%) | 103 (28.0%) |  | 1.72 [1.29-2.28] ^***^ | 1.02 [0.72-1.44] |
| 14-20 days | 204 (9.6%) | 162 (9.2%) | 42 (11.4%) |  | 1.72 [1.17-2.53] ^**^ | 0.60 [0.37-0.96] ^*^ |
| ≥ 21 days | 228 (10.7%) | 165 (9.4%) | 63 (17.1%) |  | 2.53 [1.79-3.57] ^***^ | 0.72 [0.46-1.13] |
| Missing | 192 (9.0%) | 163 (9.3%) | 29 (7.9%) |  | 1.18 [0.76-1.82] | 1.63 [0.65-4.09] |
| Food intake in the previous week |  |  |  | < 0.001 |  |  |
| More than normal or normal | 1320 (62.2%) | 1154 (65.8%) | 166 (45.1%) |  | Reference | Reference |
| A little less than normal | 219 (10.3%) | 179 (10.2%) | 40 (10.9%) |  | 1.55 [1.06-2.27] ^*^ | 1.12 [0.71-1.78] |
| Less than half of normal | 558 (26.3%) | 402 (22.9%) | 156 (42.4%) |  | 2.70 [2.11-3.45] ^***^ | 1.36 [0.98-1.88] |
| Missing | 26 (1.2%) | 20 (1.1%) | 6 (1.6%) |  | 2.09 [0.83-5.27] | 0.99 [0.32-3.08] |
| Nutritional intervention |  |  |  | < 0.001 |  |  |
| Artificial nutrition | 645 (30.4%) | 466 (26.6%) | 179 (48.6%) |  | Reference | Reference |
| Dietary nutrition | 834 (39.3%) | 752 (42.8%) | 82 (22.3%) |  | 0.28 [0.21-0.38] ^***^ | 0.50 [0.34-0.72] ^***^ |
| Multi-form of artificial and dietary nutrition | 214 (10.1%) | 180 (10.3%) | 34 (9.2%) |  | 0.49 [0.33-0.74] ^***^ | 0.62 [0.38-1.00] ^*^ |
| None | 97 (4.6%) | 78 (4.4%) | 19 (5.2%) |  | 0.63 [0.37-1.08] | 0.74 [0.39-1.38] |
| Unsure or missing | 333 (15.7%) | 279 (15.9%) | 54 (14.7%) |  | 0.50 [0.36-0.71] ^***^ | 0.69 [0.45-1.06] |
| Eating on nutritionDay |  |  |  | < 0.001 |  |  |
| Incompletely eaten | 1443 (68.0%) | 1136 (64.7%) | 307 (83.4%) |  | Reference | Reference |
| Completely eaten | 613 (28.9%) | 575 (32.8%) | 38 (10.3%) |  | 0.24 [0.17-0.35] ^***^ | 0.39 [0.26-0.58] ^***^ |
| Missing | 67 (3.2%) | 44 (2.5%) | 23 (6.3%) |  | 1.93 [1.15-3.25] ^*^ | 1.97 [0.99-3.91] |

Comparisons of patients groups between mobile patients and reduced mobility patients were performed by the Chi-square test, Fisher's exact test or Wilcoxon rank-sum test, as appropriate. *p* value, mobile patients vs. reduced mobility patients. Significant variables with *p* < 0.05 in univariate analysis were included in a logistic regression analysis to assess the association between reduced mobility and patients’ characteristics in multivariable analysis. BMI, body mass index. OR, odds ratio; CI, confidence interval; ICU, intensive care unit; LOS, length of hospital stay. * *p* < 0.05, ** *p* < 0.01, *** *p* < 0.001.

**Supplementary Table S5.** Demographic and nutritional characteristics of patients with mobility status on nutritionDay, n=5511 (Sensitivity analysis adjusted for pre-hospital functional status).

| Variable | All  n (%) | Univariate analysis | | | | Multivariable analysis |
| --- | --- | --- | --- | --- | --- | --- |
|  |  | Mobile  n (%) | Reduced mobility  n (%) | *P* value | OR [95% CI] | OR [95% CI] |
|  | 5511 | 4232 | 1279 |  |  |  |
| Number of drugs before admission |  |  |  | < 0.001 |  |  |
| None | 1663 (30.2%) | 1385 (32.7%) | 278 (21.7%) |  | Reference | Reference |
| 1-2 | 1743 (31.6%) | 1389 (32.8%) | 354 (27.7%) |  | 1.27 [1.07-1.51] ^**^ | 1.21 [1.01-1.45] ^*^ |
| 3-5 | 1136 (20.6%) | 837 (19.8%) | 299 (23.4%) |  | 1.78 [1.48-2.14] ^***^ | 1.65 [1.36-2.01] ^***^ |
| ＞5 | 629 (11.4%) | 394 (9.3%) | 235 (18.4%) |  | 2.97 [2.42-3.65] ^***^ | 2.59 [2.08-3.23] ^***^ |
| Unknown or missing | 340 (6.2%) | 227 (5.4%) | 113 (8.8%) |  | 2.48 [1.91-3.22] ^***^ | 2.09 [1.59-2.75] ^***^ |
| Weight change within last 3 months |  |  |  | < 0.001 |  |  |
| Stable weight | 2501 (45.4%) | 2051 (48.5%) | 450 (35.2%) |  | Reference | Reference |
| Weight loss | 2156 (39.1%) | 1541 (36.4%) | 615 (48.1%) |  | 1.82 [1.58-2.09] ^***^ | 1.17 [1.00-1.36] ^*^ |
| Weight gain | 320 (5.8%) | 269 (6.4%) | 51 (4.0%) |  | 0.86 [0.63-1.19] | 0.72 [0.52-1.01] |
| Unsure or missing | 534 (9.7%) | 371 (8.8%) | 163 (12.7%) |  | 2.00 [1.62-2.47] ^***^ | 1.80 [1.44-2.25] ^***^ |
| Food intake in the previous week |  |  |  | < 0.001 |  |  |
| More than normal or normal | 3661 (66.4%) | 3002 (70.9%) | 659 (51.5%) |  | Reference | Reference |
| A little less than normal | 707 (12.8%) | 492 (11.6%) | 215 (16.8%) |  | 1.99 [1.66-2.39] ^***^ | 1.50 [1.24-1.82] ^***^ |
| Less than half of normal | 1104 (20.0%) | 706 (16.7%) | 398 (31.1%) |  | 2.57 [2.21-2.98] ^***^ | 1.45 [1.23-1.72] ^***^ |
| Missing | 39 (0.7%) | 32 (0.8%) | 7 (0.5%) |  | 1.00 [0.44-2.27] | 0.47 [0.20-1.11] |
| Nutritional intervention |  |  |  | < 0.001 |  |  |
| Artificial nutrition | 1280 (23.2%) | 813 (19.2%) | 467 (36.5%) |  | Reference | Reference |
| Dietary nutrition | 2877 (52.2%) | 2393 (56.5%) | 484 (37.8%) |  | 0.35 [0.30-0.41] ^***^ | 0.49 [0.41-0.58] ^***^ |
| Multi-form of artificial and dietary nutrition | 569 (10.3%) | 408 (9.6%) | 161 (12.6%) |  | 0.69 [0.55-0.85] ^***^ | 0.83 [0.66-1.04] |
| None | 175 (3.2%) | 137 (3.2%) | 38 (3.0%) |  | 0.48 [0.33-0.70] ^***^ | 0.62 [0.42-0.92] ^*^ |
| Unsure or missing | 610 (11.1%) | 481 (11.4%) | 129 (10.1%) |  | 0.47 [0.37-0.58] ^***^ | 0.56 [0.44-0.70] ^***^ |
| Eating on nutritionDay |  |  |  | < 0.001 |  |  |
| Incompletely eaten | 3252 (59.0%) | 2285 (54.0%) | 967 (75.6%) |  | Reference | Reference |
| Completely eaten | 2087 (37.9%) | 1832 (43.3%) | 255 (19.9%) |  | 0.33 [0.28-0.38] ^***^ | 0.45 [0.39-0.54] ^***^ |
| Missing | 172 (3.1%) | 115 (2.7%) | 57 (4.5%) |  | 1.17 [0.85-1.62] | 1.01 [0.72-1.43] |

Comparisons of patients groups between mobile patients and reduced mobility patients were performed by the Chi-square test, Fisher's exact test or Wilcoxon rank-sum test, as appropriate. *p* value, mobile patients vs. reduced mobility patients. Significant variables with *p* < 0.05 in univariate analysis were included in a logistic regression analysis to assess the association between reduced mobility and patients’ characteristics in multivariable analysis. OR, odds ratio; CI, confidence interval. * *p* < 0.05, ** *p* < 0.01, *** *p* < 0.001.

**Supplementary Table S6.** Demographic and nutritional characteristics of patients with mobility status on nutritionDay, n=5511 (Sensitivity analysis adjusted for underlying comorbidities).

| Variable | All  n (%) | Univariate analysis | | | | Multivariable analysis |
| --- | --- | --- | --- | --- | --- | --- |
|  |  | Mobile  n (%) | Reduced mobility  n (%) | *P* value | OR [95% CI] | OR [95% CI] |
|  | 5511 | 4232 | 1279 |  |  |  |
| Comorbidity |  |  |  | < 0.001 |  |  |
| No | 1785 (32.4%) | 1474 (34.8%) | 311 (24.3%) |  |  | Reference |
| Yes | 3218 (58.4%) | 2366 (55.9%) | 852 (66.6%) |  | 1.71 [1.48-1.97] ^***^ | 1.85 [1.60-2.16] ^***^ |
| Missing | 508 (9.2%) | 392 (9.3%) | 116 (9.1%) |  | 1.40 [1.10-1.78] ^**^ | 1.57 [1.22-2.01] ^***^ |
| Nutritional intervention |  |  |  | < 0.001 |  |  |
| Artificial nutrition | 1280 (23.2%) | 813 (19.2%) | 467 (36.5%) |  | Reference | Reference |
| Dietary nutrition | 2877 (52.2%) | 2393 (56.5%) | 484 (37.8%) |  | 0.35 [0.30-0.41] ^***^ | 0.46 [0.39-0.54] ^***^ |
| Multi-form of artificial and dietary nutrition | 569 (10.3%) | 408 (9.6%) | 161 (12.6%) |  | 0.69 [0.55-0.85] ^***^ | 0.81 [0.64-1.01] |
| None | 175 (3.2%) | 137 (3.2%) | 38 (3.0%) |  | 0.48 [0.33-0.70] ^***^ | 0.53 [0.36-0.78] ^**^ |
| Unsure or missing | 610 (11.1%) | 481 (11.4%) | 129 (10.1%) |  | 0.47 [0.37-0.58] ^***^ | 0.53 [0.42-0.67] ^***^ |
| Eating on nutritionDay |  |  |  | < 0.001 |  |  |
| Incompletely eaten | 3252 (59.0%) | 2285 (54.0%) | 967 (75.6%) |  | Reference | Reference |
| Completely eaten | 2087 (37.9%) | 1832 (43.3%) | 255 (19.9%) |  | 0.33 [0.28-0.38] ^***^ | 0.38 [0.33-0.45] ^***^ |
| Missing | 172 (3.1%) | 115 (2.7%) | 57 (4.5%) |  | 1.17 [0.85-1.62] | 0.99 [0.71-1.39] |

Comparisons of patients groups between mobile patients and reduced mobility patients were performed by the Chi-square test, Fisher's exact test or Wilcoxon rank-sum test, as appropriate. *p* value, mobile patients vs. reduced mobility patients. Significant variables with *p* < 0.05 in univariate analysis were included in a logistic regression analysis to assess the association between reduced mobility and patients’ characteristics in multivariable analysis. OR, odds ratio; CI, confidence interval. * *p* < 0.05, ** *p* < 0.01, *** *p* < 0.001.

| (A) Mobility status in patients from 2010 to 2015, n =1590 | (B) Mobility status in patients from 2016 to 2020, n=3921 |
| --- | --- |
| 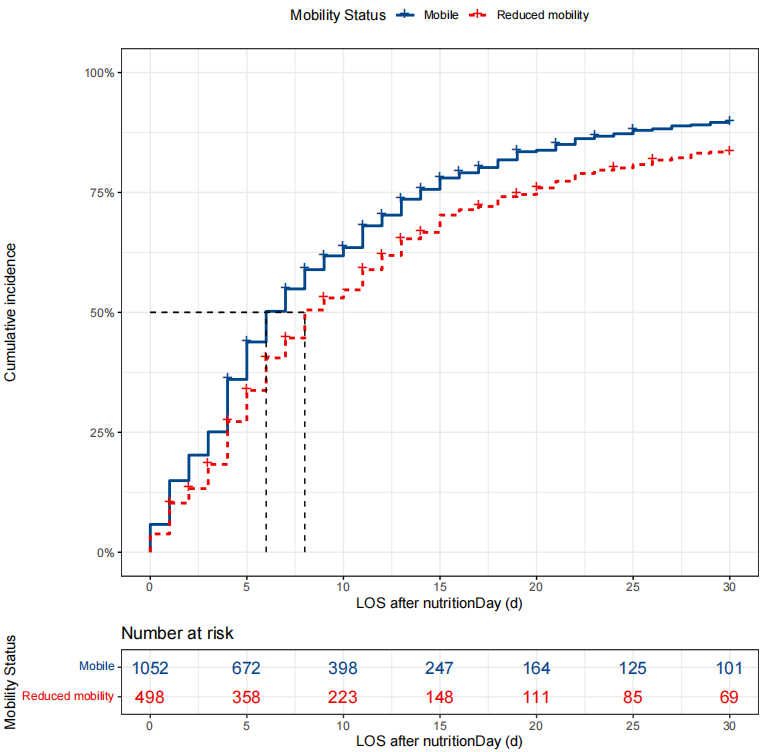 | 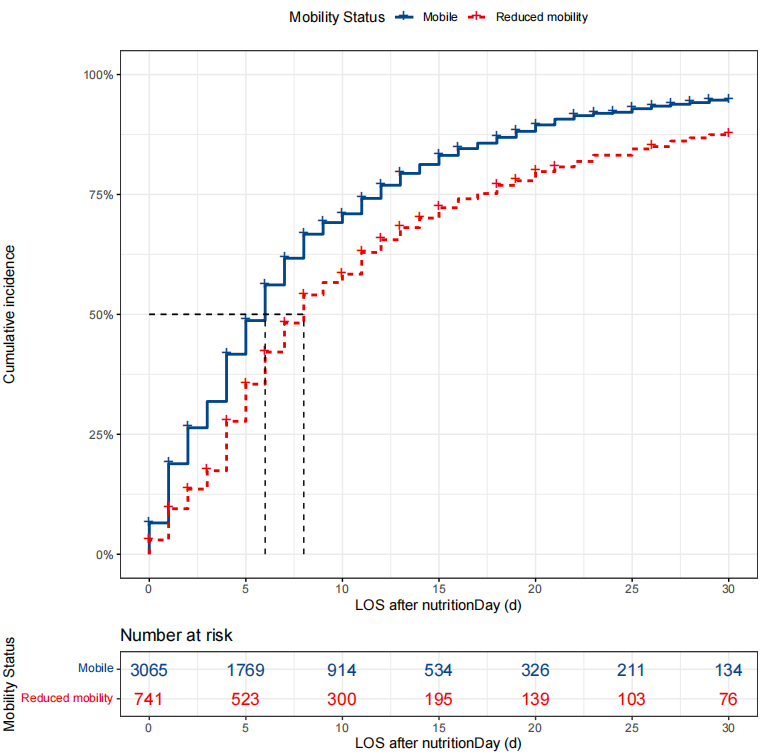 |
| LOS after nutritionDay: median (95% CI)  Patients with mobile vs. Patients with reduced mobility: 6 days (6-7) vs. 8 days (8-10), *p* < 0.001. | LOS after nutritionDay: median (95% CI)  Patients with mobile vs. Patients with reduced mobility: 6 days (5-6) vs. 8 days (7-8), *p* < 0.001. |
| (C) Mobility status in all patients from 2010 to 2020, n = 5511 | (D) Mobility status in eastern region, n = 4994 |
| 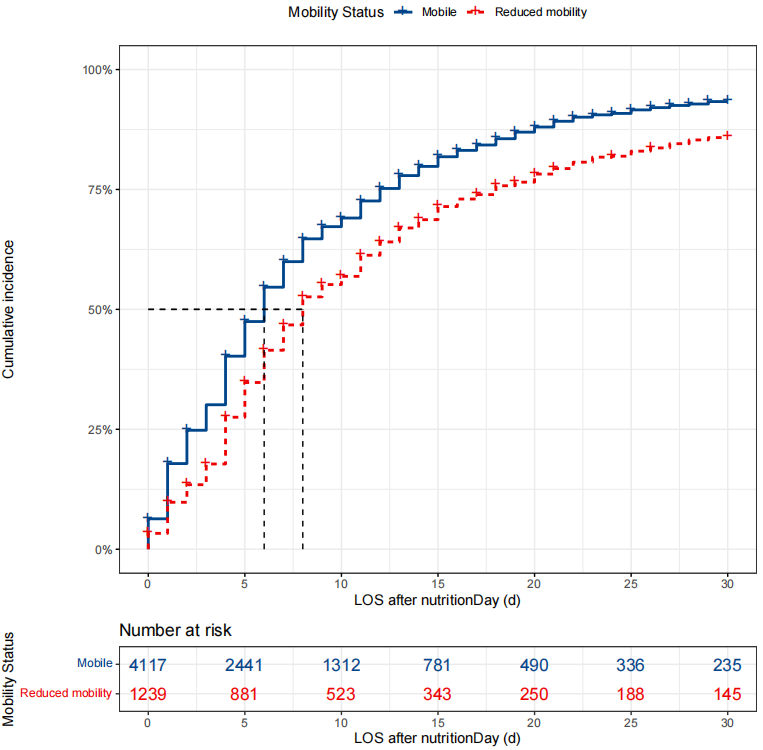 | 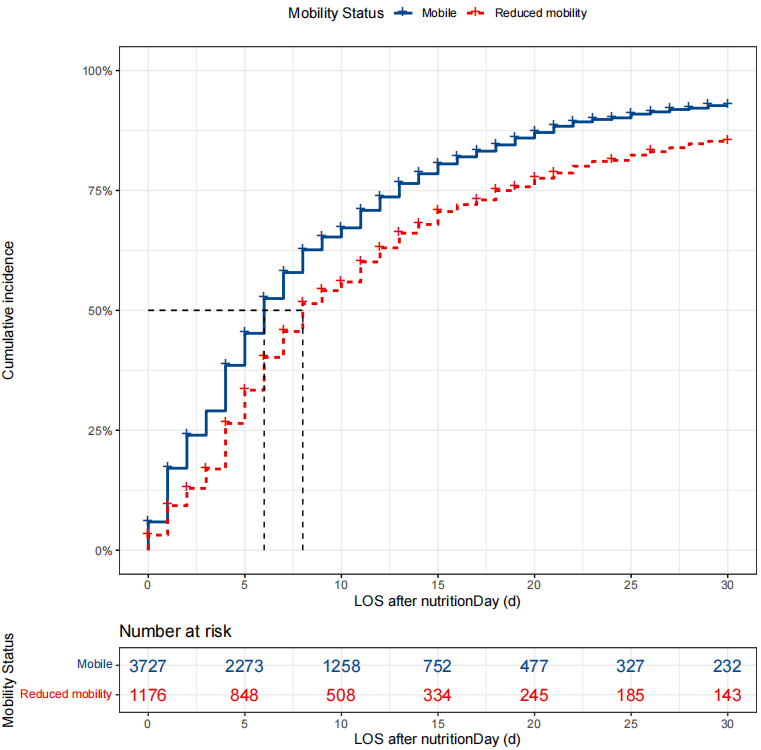 |
| LOS after nutritionDay: median (95% CI)  Patients with mobile vs. Patients with reduced mobility: 6 days (6-6) vs. 8 days (8-9), *p* < 0.001. | LOS after nutritionDay: median (95% CI)  Patients with mobile vs. Patients with reduced mobility: 6 days (6-6) vs. 8 days (8-9), *p* < 0.001. |
| (E) Mobility status in western region, n = 517 |  |
| 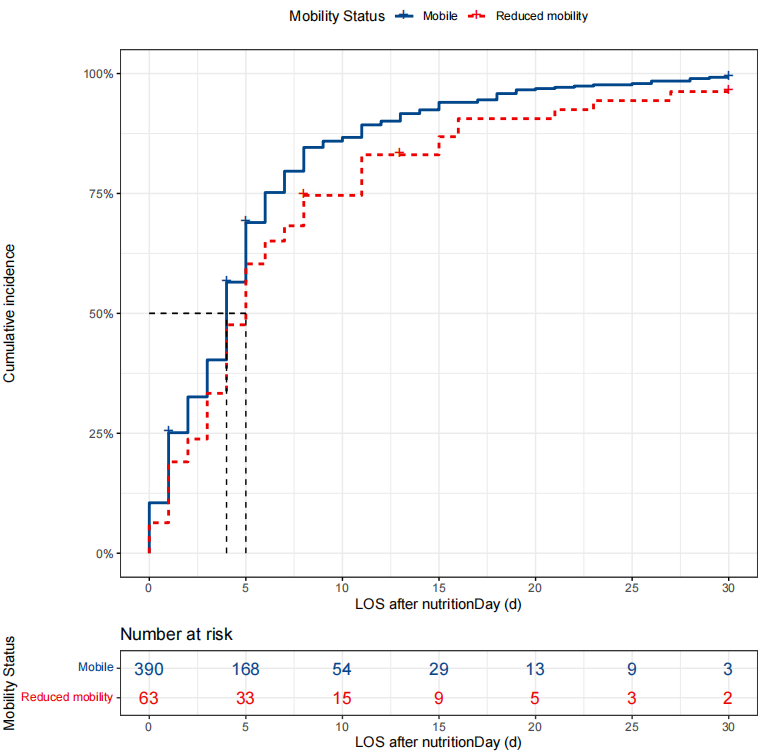 |  |
| LOS after nutritionDay: median (95% CI)  Patients with mobile vs. Patients with reduced mobility: 4 days (4-4) vs. 5 days (4-6), *p* < 0.05. |  |

**Supplementary Figure S1.** Cumulative incidence of discharged home within 30 days after nutritionDay in patients with different nutritional interventions and meal eaten stratified by survey years and regions. Missing data were excluded. Differences in median (95% CI) LOS after nutritionDay between groups were tested using the log-rank test. LOS, length of hospital stay; CI, confidence interval. **(A)** Mobility status in patients from 2010 to 2015, n = 1590. **(B)** Mobility status in patients from 2016 to 2020, n = 3921. **(C)** Mobility status in all patients from 2010 to 2020, n = 5511. **(D)** Mobility status in patients distributed in eastern region, n = 4994. **(E)** Mobility status in patients distributed in western region, n = 517.


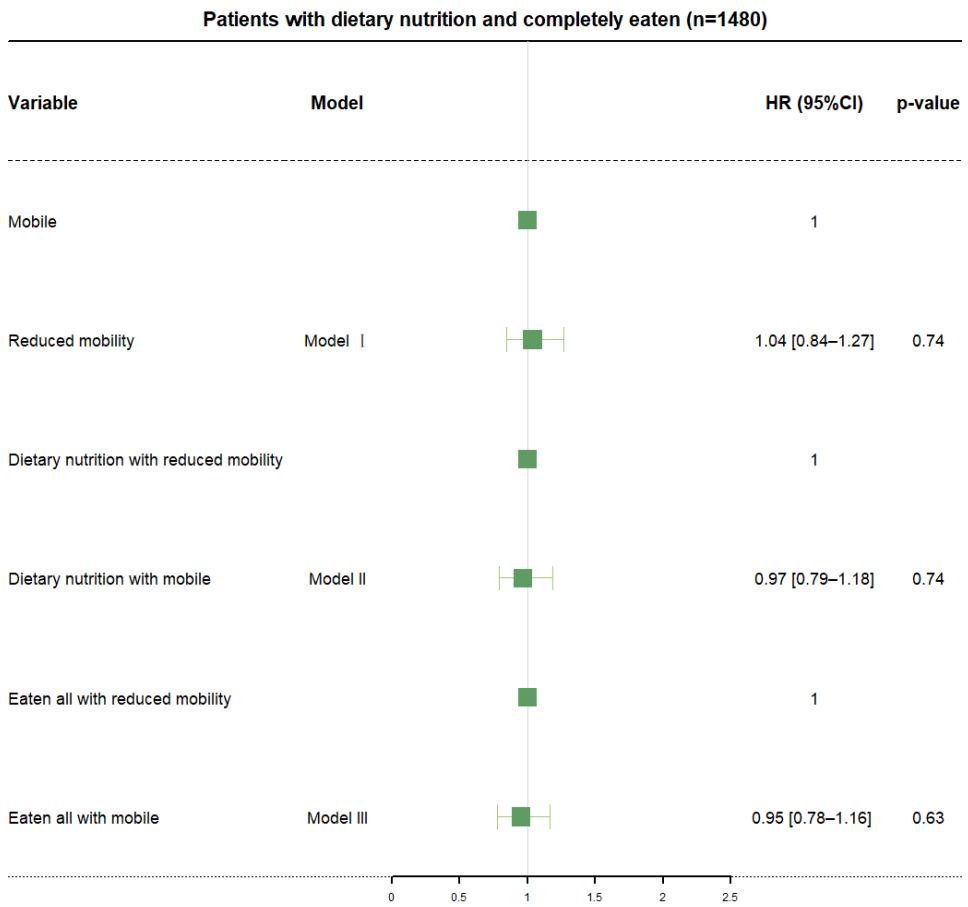


**Supplementary Figure S2.** Subgroup analysis of associations of mobility, nutritional interventions, and meal eaten with discharged home in patients with dietary nutrition and completely eaten**.** Cox regression models with HRs were used to analyze discharged home. Model I: Multivariable analysis with nutritional intervention, meal eaten and mobility status. Model II: Meal eaten and combined nutritional intervention with mobility status added to the multivariable analysis. Model III: Nutritional intervention and combined meal eaten with mobility status added to the multivariable analysis. All data are presented as HR and 95% CI. HR, hazard ratio; CI, confidence interval.


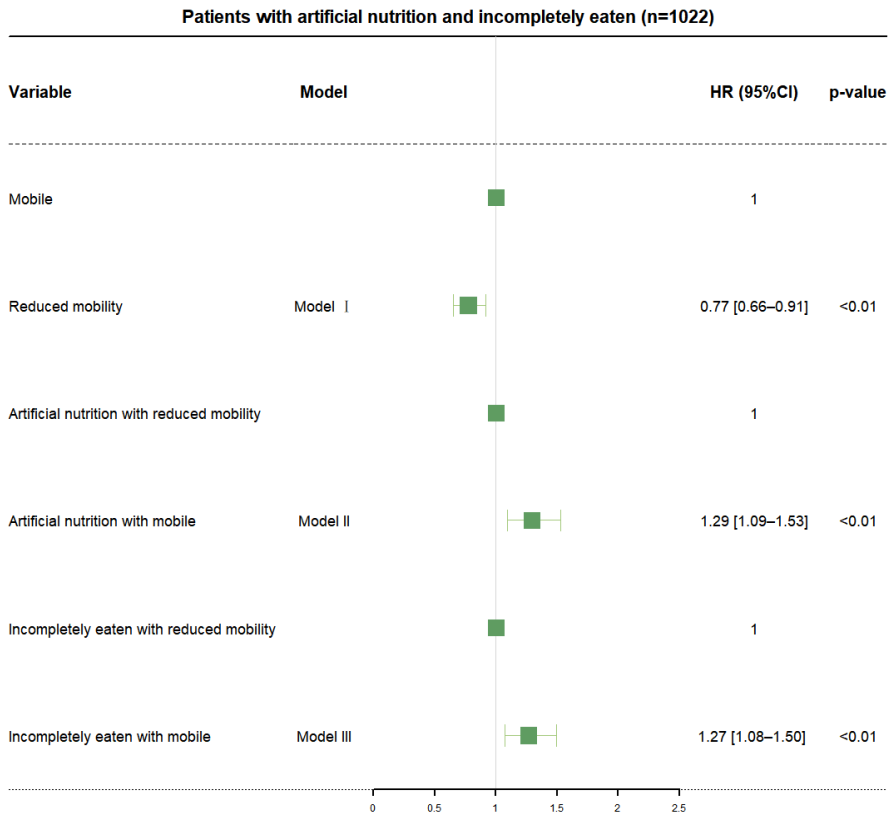


**Supplementary Figure S3.** Subgroup analysis of associations of mobility, nutritional interventions, and meal eaten with discharged home in patients with artificial nutrition and incompletely eaten**.** Cox regression models with HRs were used to analyze discharged home. Model I: Multivariable analysis with nutritional intervention, meal eaten and mobility status. Model II: Meal eaten and combined nutritional intervention with mobility status added to the multivariable analysis. Model III: Nutritional intervention and combined meal eaten with mobility status added to the multivariable analysis. All data are presented as HR and 95% CI. HR, hazard ratio; CI, confidence interval.


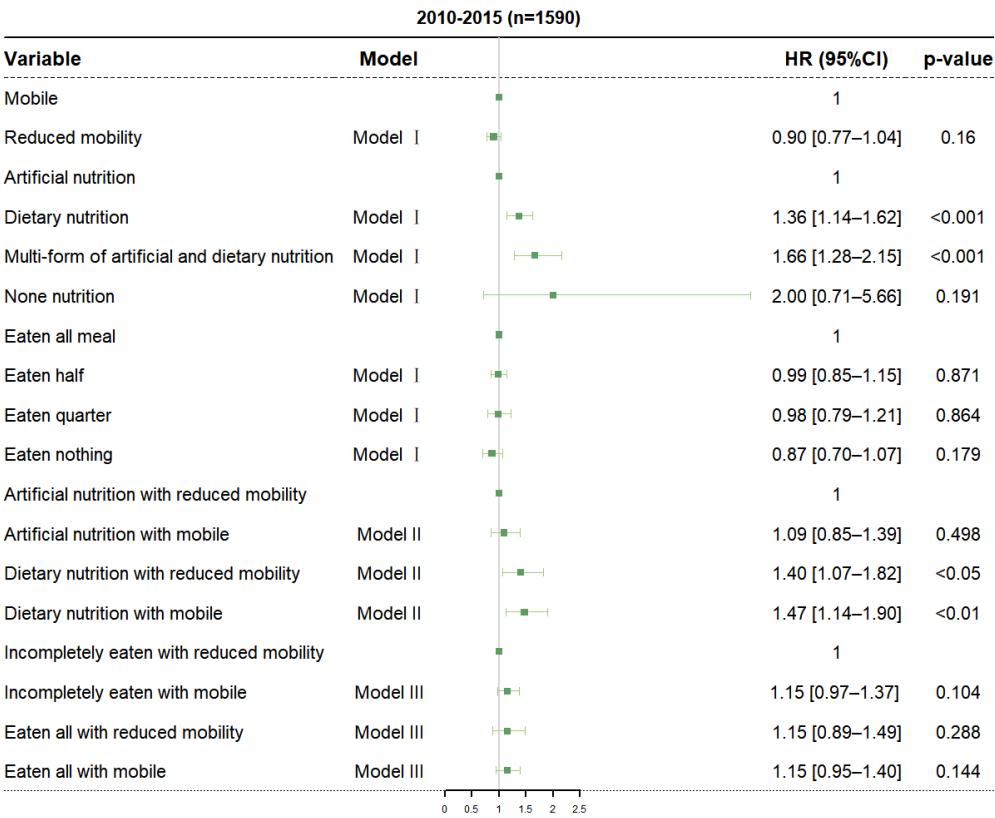


**Supplementary Figure S4.** Subgroup analysis of associations of mobility, nutritional interventions, and meal eaten with discharged home in patients from 2010 to 2015**.** Cox regression models with HRs were used to analyze discharged home. Model I: Multivariable analysis with nutritional intervention, meal eaten and mobility status. Model II: Meal eaten and combined nutritional intervention with mobility status added to the multivariable analysis. Model III: Nutritional intervention and combined meal eaten with mobility status added to the multivariable analysis. All data are presented as HR and 95% CI. HR, hazard ratio; CI, confidence interval.


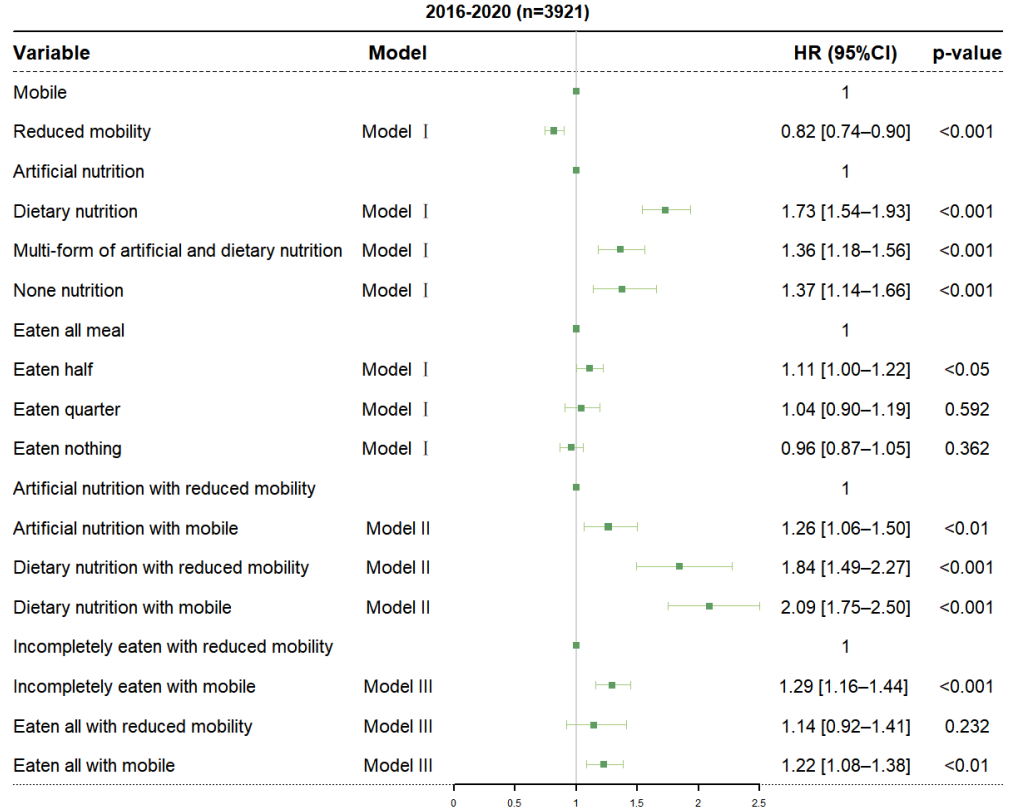


**Supplementary Figure S5.** Subgroup analysis of associations of mobility, nutritional interventions, and meal eaten with discharged home in patients from 2016 to 2020**.** Cox regression models with HRs were used to analyze discharged home. Model I: Multivariable analysis with nutritional intervention, meal eaten and mobility status. Model II: Meal eaten and combined nutritional intervention with mobility status added to the multivariable analysis. Model III: Nutritional intervention and combined meal eaten with mobility status added to the multivariable analysis. All data are presented as HR and 95% CI. HR, hazard ratio; CI, confidence interval.


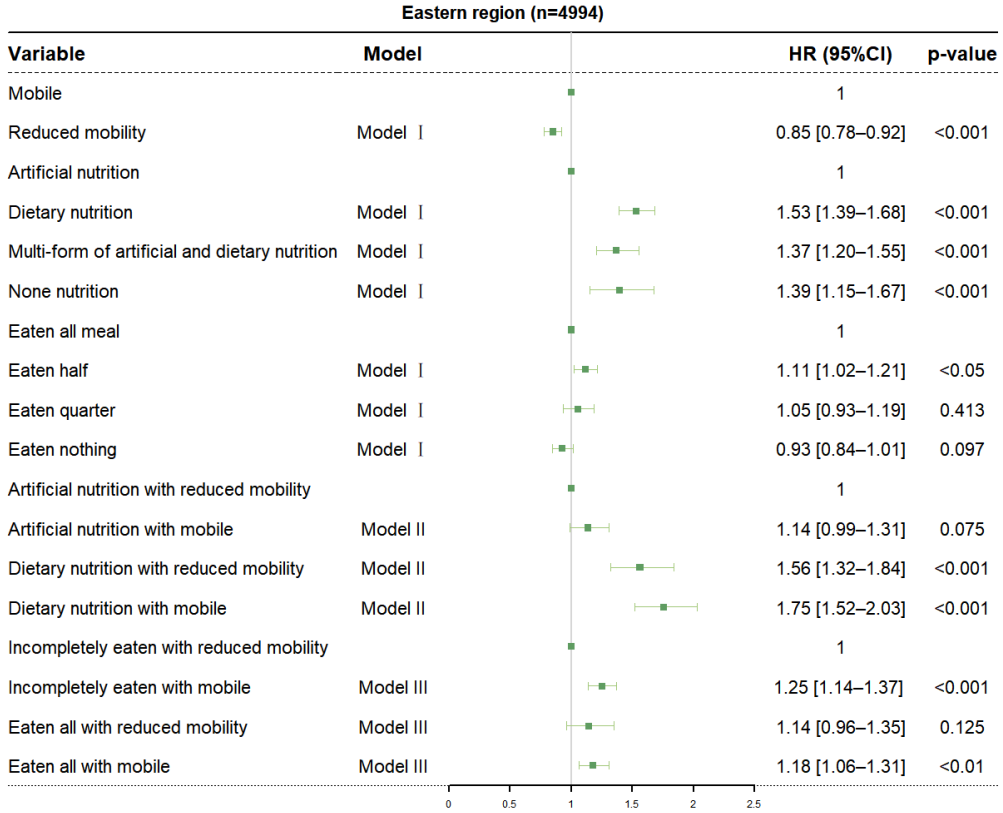


**Supplementary Figure S6.** Subgroup analysis of associations of mobility, nutritional interventions, and meal eaten with discharged home in patients in eastern region**.** Cox regression models with HRs were used to analyze discharged home. Model I: Multivariable analysis with nutritional intervention, meal eaten and mobility status. Model II: Meal eaten and combined nutritional intervention with mobility status added to the multivariable analysis. Model III: Nutritional intervention and combined meal eaten with mobility status added to the multivariable analysis. All data are presented as HR and 95% CI. HR, hazard ratio; CI, confidence interval.


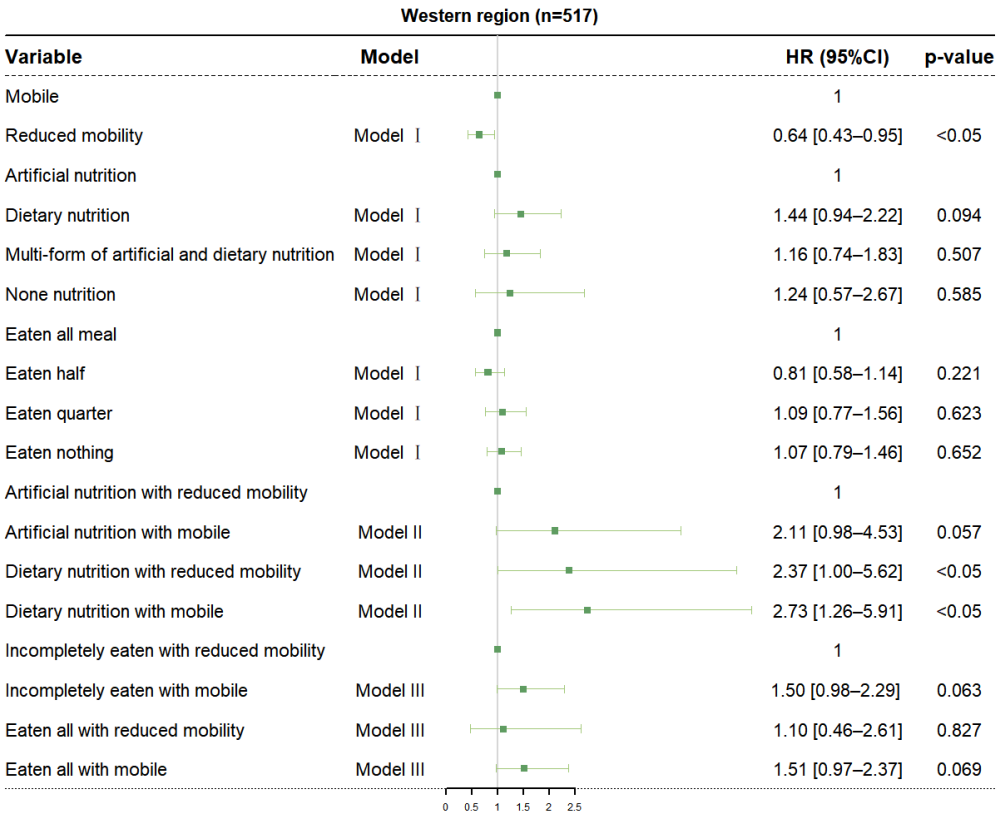


**Supplementary Figure S7.** Subgroup analysis of associations of mobility, nutritional interventions, and meal eaten with discharged home in patients in western region**.** Cox regression models with HRs were used to analyze discharged home. Model I: Multivariable analysis with nutritional intervention, meal eaten and mobility status. Model II: Meal eaten and combined nutritional intervention with mobility status added to the multivariable analysis. Model III: Nutritional intervention and combined meal eaten with mobility status added to the multivariable analysis. All data are presented as HR and 95% CI. HR, hazard ratio; CI, confidence interval.


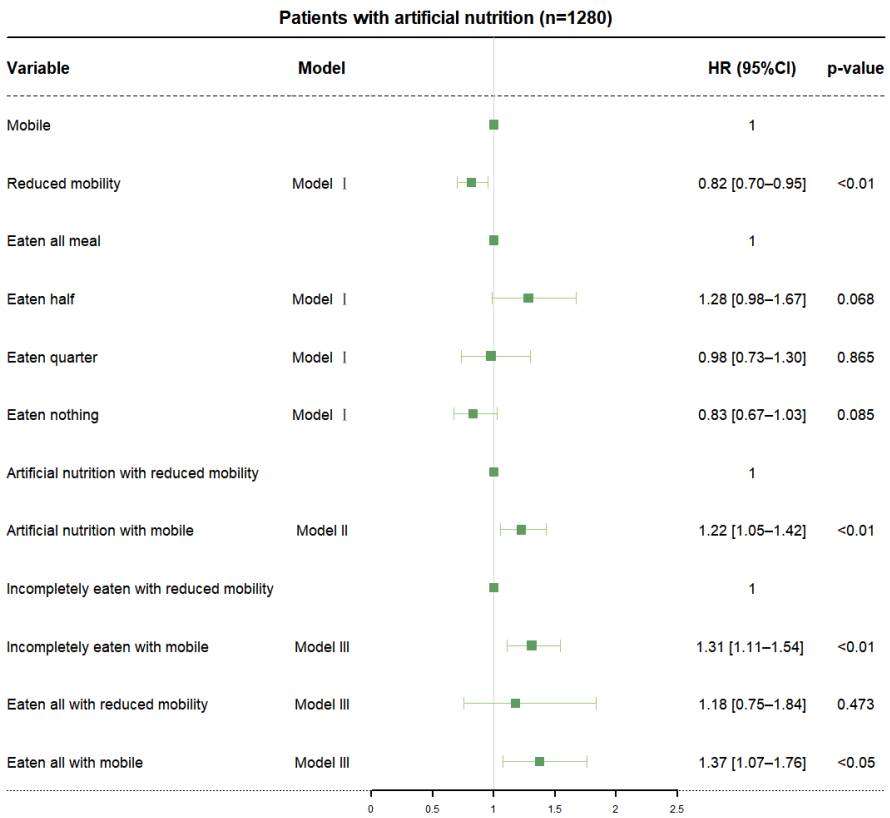


**Supplementary Figure S8.** Subgroup analysis of associations of mobility, nutritional interventions, and meal eaten with discharged home in patients with artificial nutrition**.** Cox regression models with HRs were used to analyze discharged home. Model I: Multivariable analysis with nutritional intervention, meal eaten and mobility status. Model II: Meal eaten and combined nutritional intervention with mobility status added to the multivariable analysis. Model III: Nutritional intervention and combined meal eaten with mobility status added to the multivariable analysis. All data are presented as HR and 95% CI. HR, hazard ratio; CI, confidence interval.


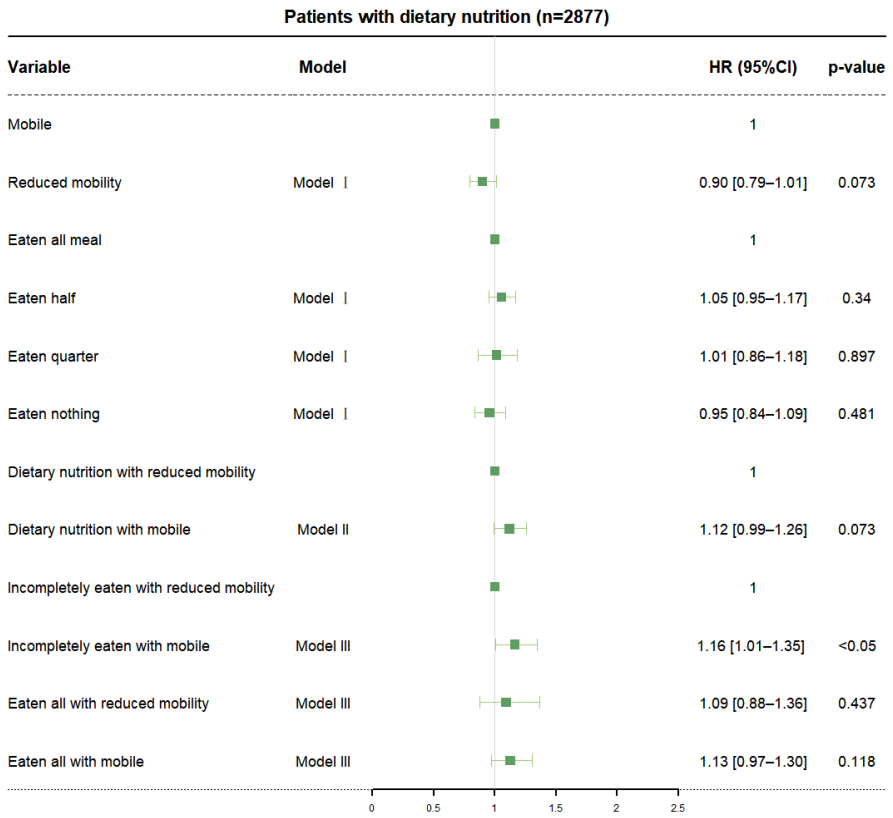


**Supplementary Figure S9.** Subgroup analysis of associations of mobility, nutritional interventions, and meal eaten with discharged home in patients with dietary nutrition**.** Cox regression models with HRs were used to analyze discharged home. Model I: Multivariable analysis with nutritional intervention, meal eaten and mobility status. Model II: Meal eaten and combined nutritional intervention with mobility status added to the multivariable analysis. Model III: Nutritional intervention and combined meal eaten with mobility status added to the multivariable analysis. All data are presented as HR and 95% CI. HR, hazard ratio; CI, confidence interval.


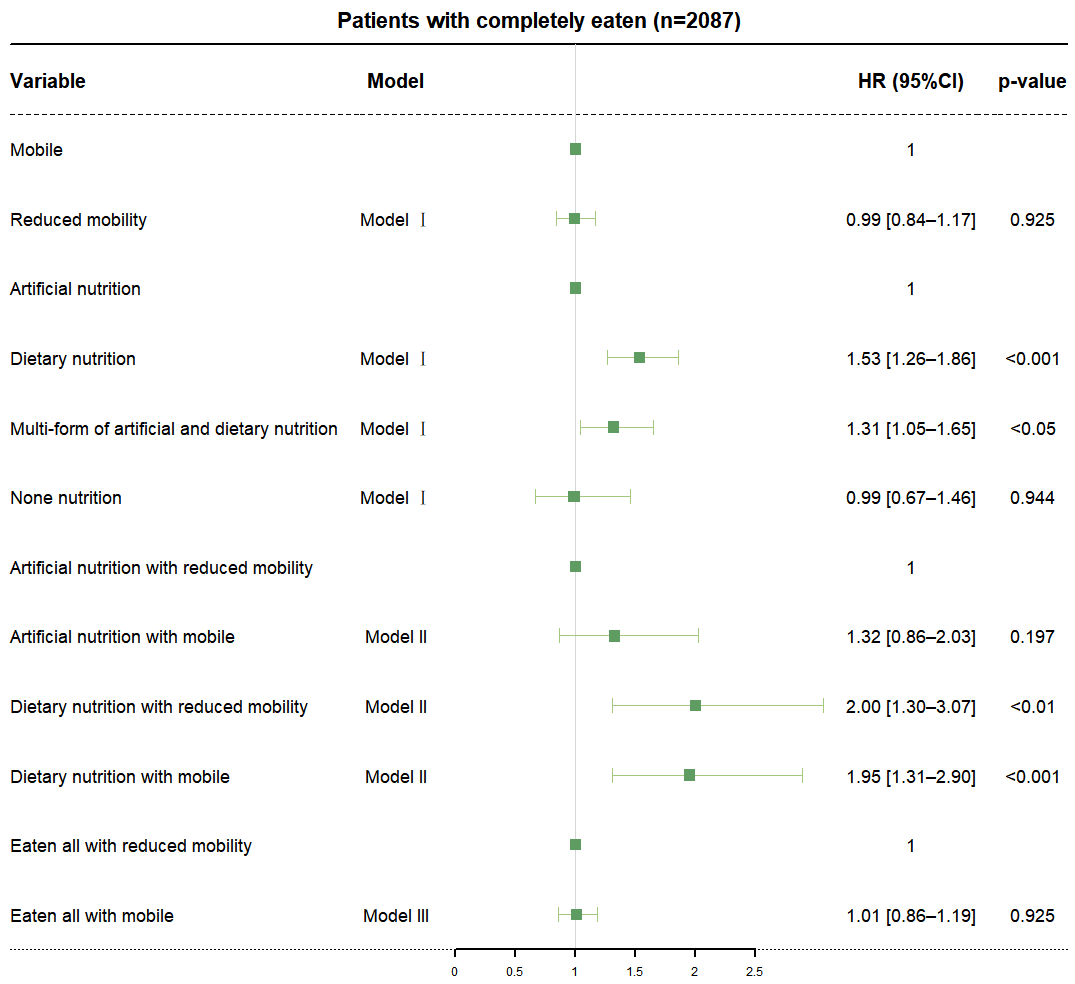


**Supplementary Figure S10.** Subgroup analysis of associations of mobility, nutritional interventions, and meal eaten with discharged home in patients with completely eaten**.** Cox regression models with HRs were used to analyze discharged home. Model I: Multivariable analysis with nutritional intervention, meal eaten and mobility status. Model II: Meal eaten and combined nutritional intervention with mobility status added to the multivariable analysis. Model III: Nutritional intervention and combined meal eaten with mobility status added to the multivariable analysis. All data are presented as HR and 95% CI. HR, hazard ratio; CI, confidence interval.


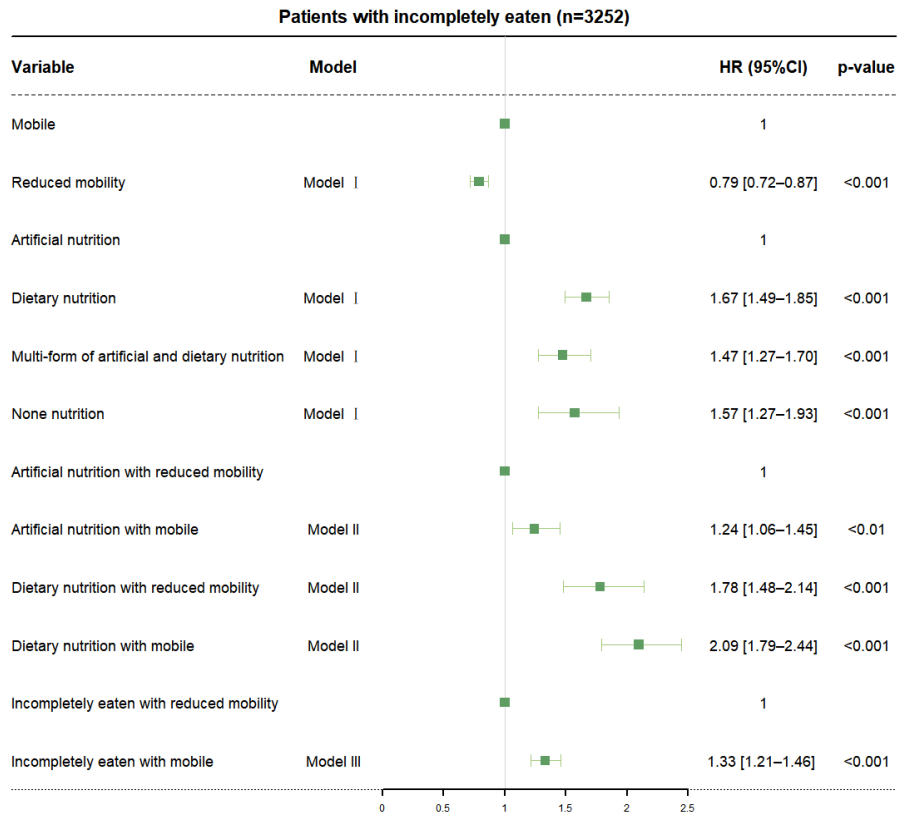


**Supplementary Figure S11.** Subgroup analysis of associations of mobility, nutritional interventions, and meal eaten with discharged home in patients with incompletely eaten**.** Cox regression models with HRs were used to analyze discharged home. Model I: Multivariable analysis with nutritional intervention, meal eaten and mobility status. Model II: Meal eaten and combined nutritional intervention with mobility status added to the multivariable analysis. Model III: Nutritional intervention and combined meal eaten with mobility status added to the multivariable analysis. All data are presented as HR and 95% CI. HR, hazard ratio; CI, confidence interval.


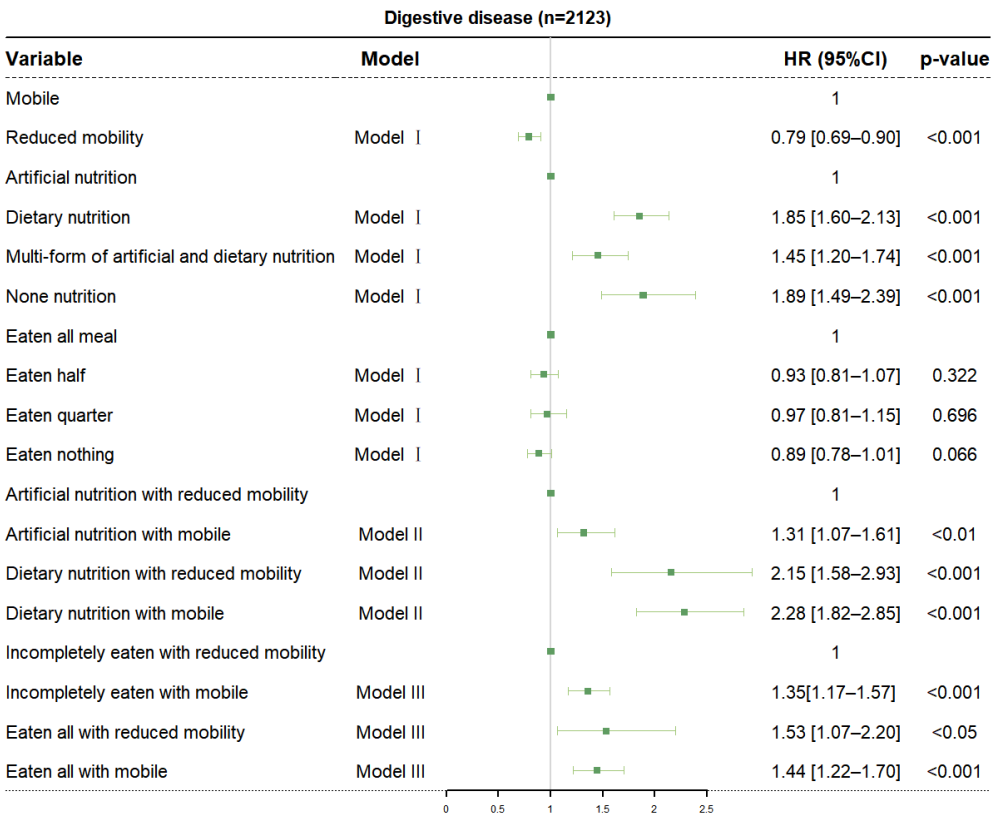


**Supplementary Figure S12.** Sensitivity analysis of associations of mobility, nutritional interventions, and meal eaten with discharged home based on digestive disease of primary diagnosis**.** Cox regression models with HRs were used to analyze discharged home. Model I: Multivariable analysis with nutritional intervention, meal eaten and mobility status. Model II: Meal eaten and combined nutritional intervention with mobility status added to the multivariable analysis. Model III: Nutritional intervention and combined meal eaten with mobility status added to the multivariable analysis. All data are presented as HR and 95% CI. HR, hazard ratio; CI, confidence interval.


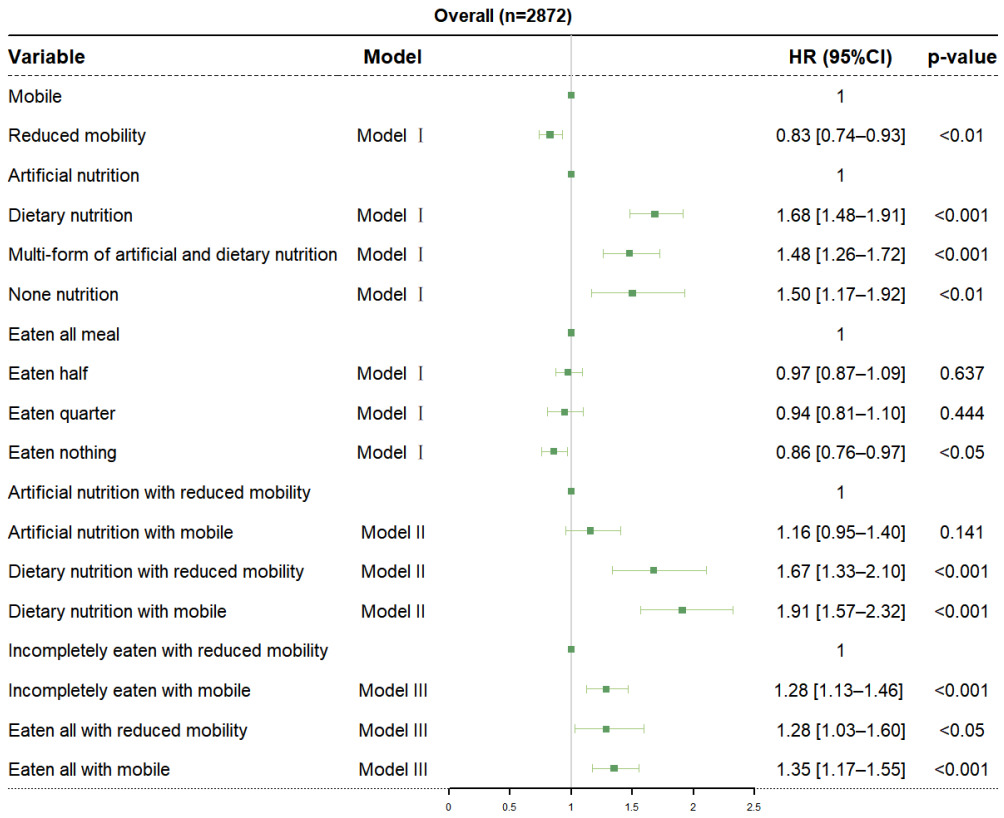


**Supplementary Figure S13.** Sensitivity analysis of associations of mobility, nutritional interventions, and meal eaten with discharged home with the exclusion of missing values**.** Cox regression models with HRs were used to analyze discharged home. Model I: Multivariable analysis with nutritional intervention, meal eaten and mobility status. Model II: Meal eaten and combined nutritional intervention with mobility status added to the multivariable analysis. Model III: Nutritional intervention and combined meal eaten with mobility status added to the multivariable analysis. All data are presented as HR and 95% CI. HR, hazard ratio; CI, confidence interval.


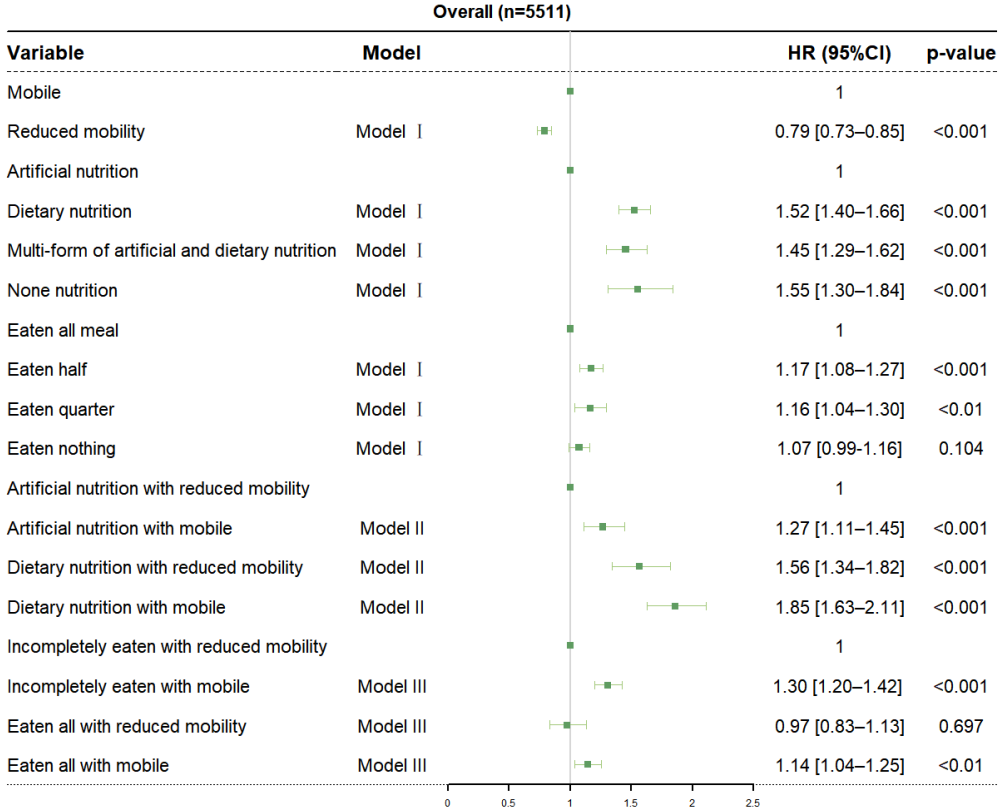


**Supplementary Figure S14.** Sensitivity analysis of associations of mobility, nutritional interventions, and meal eaten with discharged home adjusted for pre-hospital functional status**.** Cox regression models with HRs were used to analyze discharged home. Model I: Multivariable analysis with nutritional intervention, meal eaten and mobility status. Model II: Meal eaten and combined nutritional intervention with mobility status added to the multivariable analysis. Model III: Nutritional intervention and combined meal eaten with mobility status added to the multivariable analysis. All data are presented as HR and 95% CI. HR, hazard ratio; CI, confidence interval.


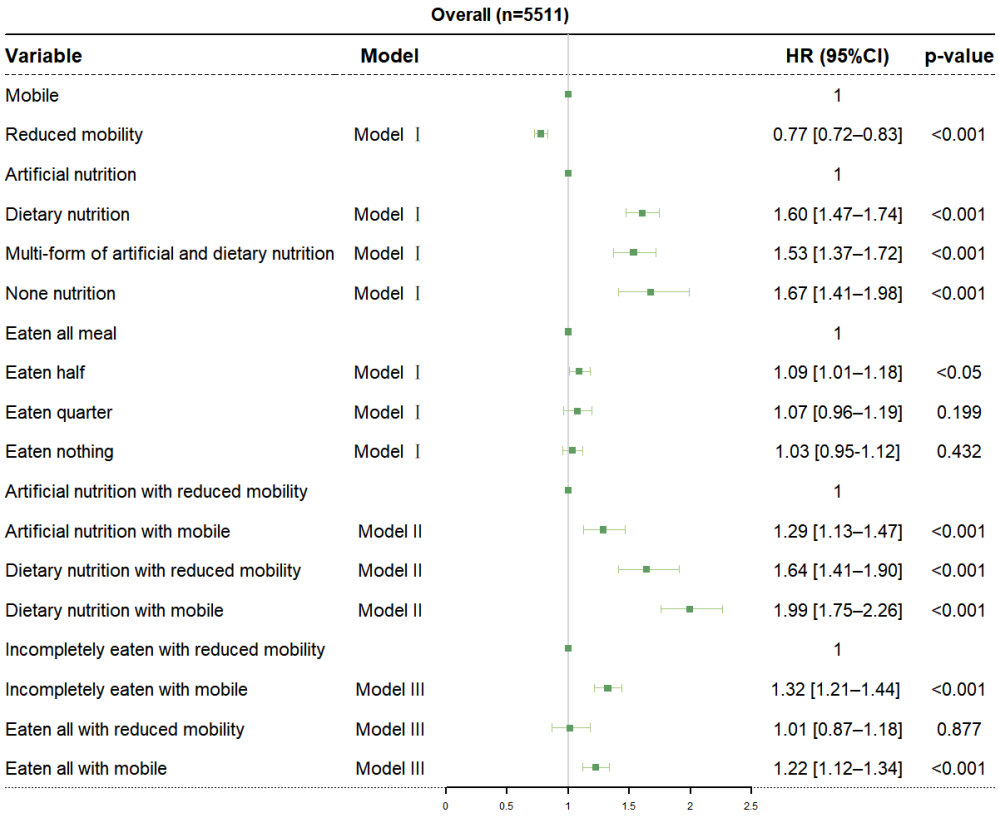


**Supplementary Figure S15.** Sensitivity analysis of associations of mobility, nutritional interventions, and meal eaten with discharged home adjusted for underlying comorbidities**.** Cox regression models with HRs were used to analyze discharged home. Model II: Meal eaten and combined nutritional intervention with mobility status added to the multivariable analysis. Model III: Nutritional intervention and combined meal eaten with mobility status added to the multivariable analysis. All data are presented as HR and 95% CI. HR, hazard ratio; CI, confidence interval.

| (A) Mobility status in patients with artificial nutrition, n=696 | (B) Mobility status in patients with dietary nutrition, n=1721 |
| --- | --- |
| 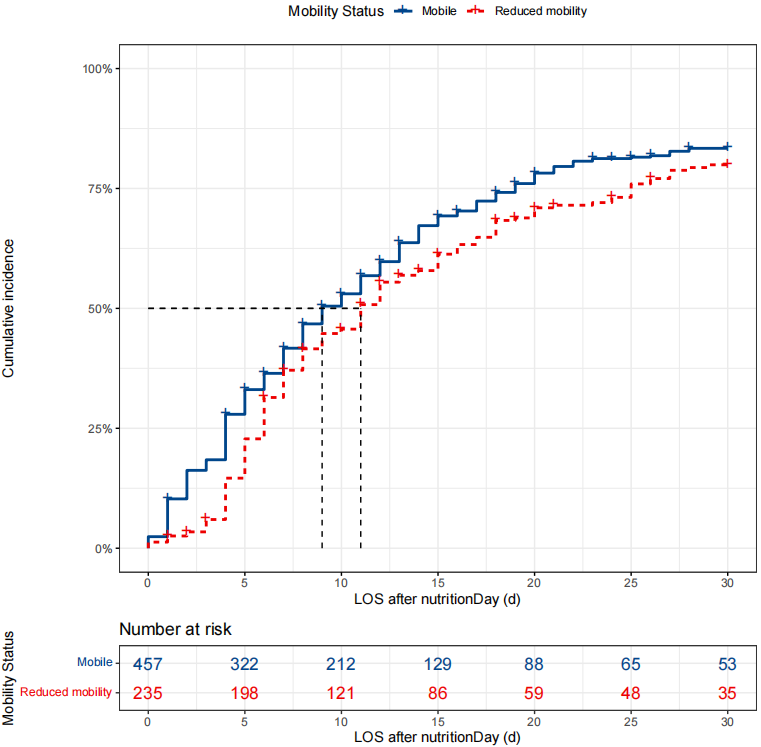 | 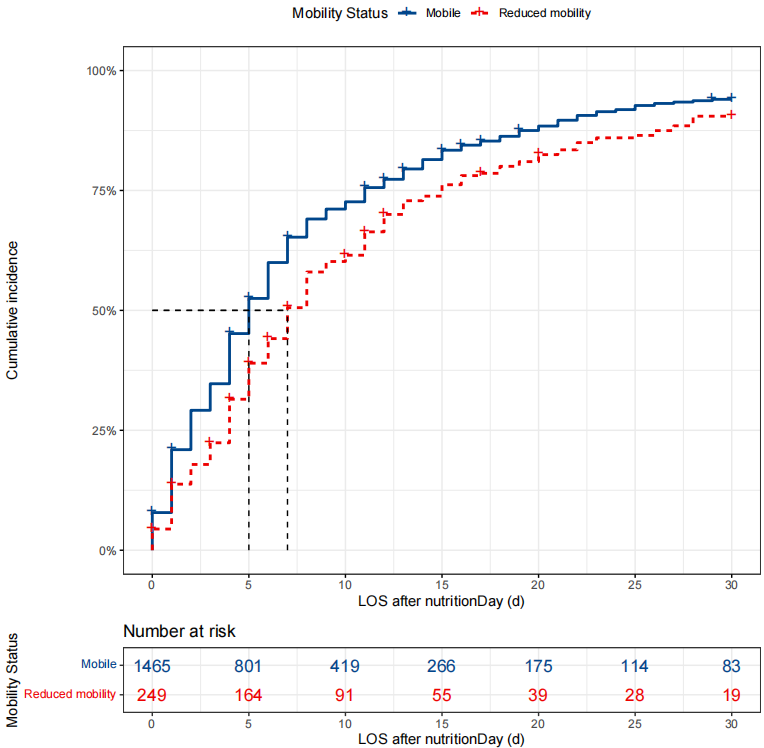 |
| LOS after nutritionDay: median (95% CI)  Patients with mobile vs. Patients with reduced mobility: 9 days (8-11) vs. 11 days (9-14), *p* < 0.05. | LOS after nutritionDay: median (95% CI)  Patients with mobile vs. Patients with reduced mobility: 5 days (5-6) vs. 7 days (6-8), *p* < 0.001. |
| (C) Mobility status in patients with completely eaten, n=1240 | (D) Mobility status in patients with incompletely eaten, n=1632 |
| 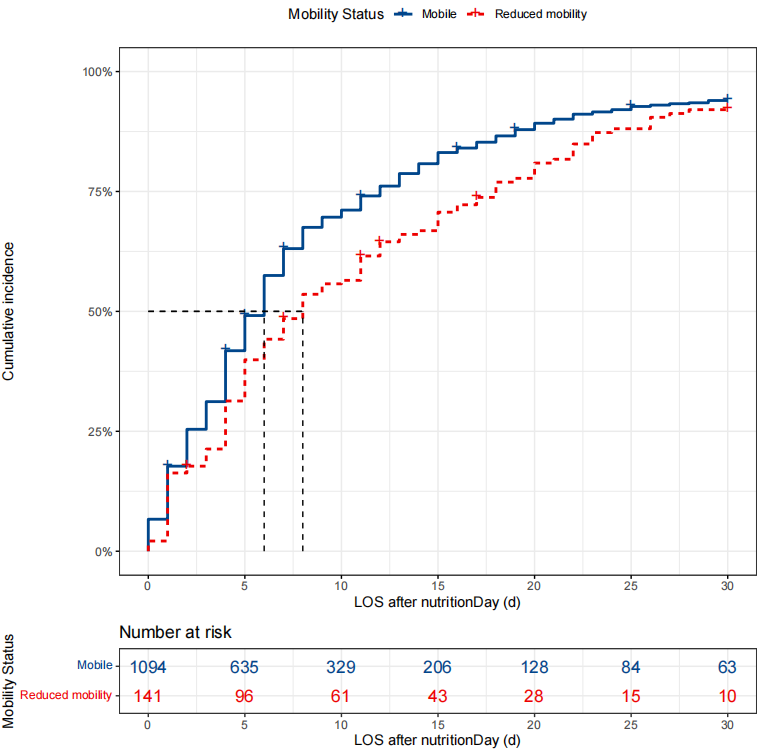 | 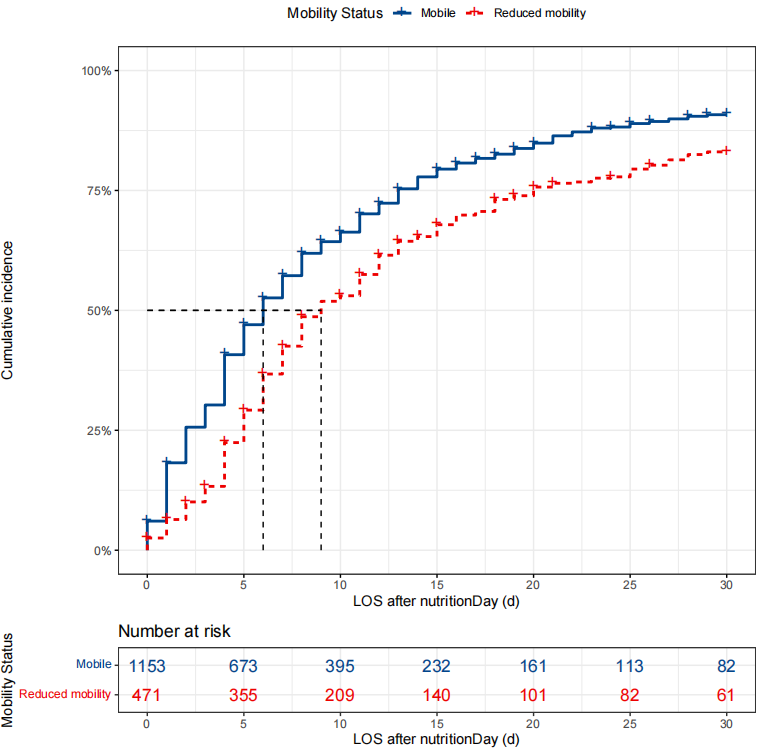 |
| LOS after nutritionDay: median (95% CI)  Patients with mobile vs. Patients with reduced mobility: 6 days (5-6) vs. 8 days (6-11), *p* < 0.01. | LOS after nutritionDay: median (95% CI)  Patients with mobile vs. Patients with reduced mobility: 6 days (6-7) vs. 9 days (8-11), *p* < 0.001. |
| (E) Mobility status in digestive disease patients with artificial nutrition, n=645 | (F) Mobility status in digestive disease patients with incompletely eaten, n=1443 |
| 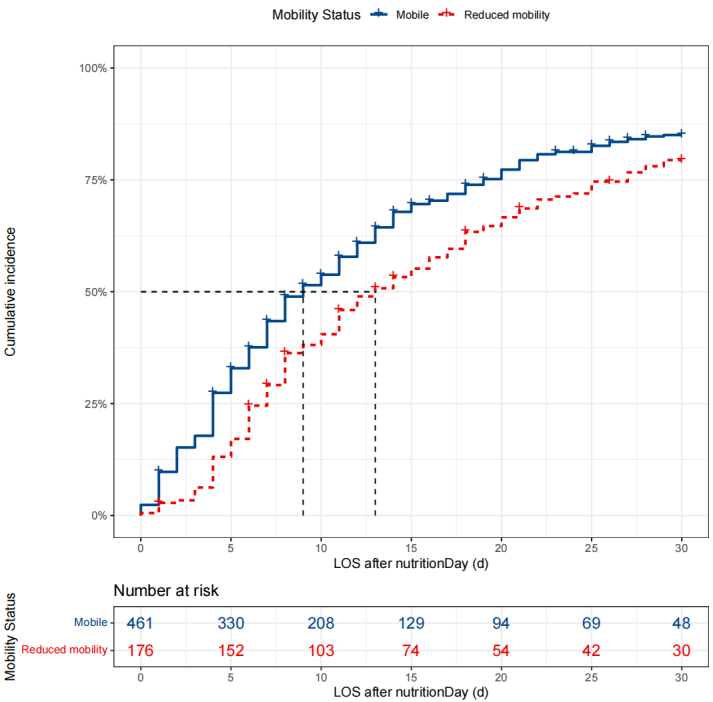 | 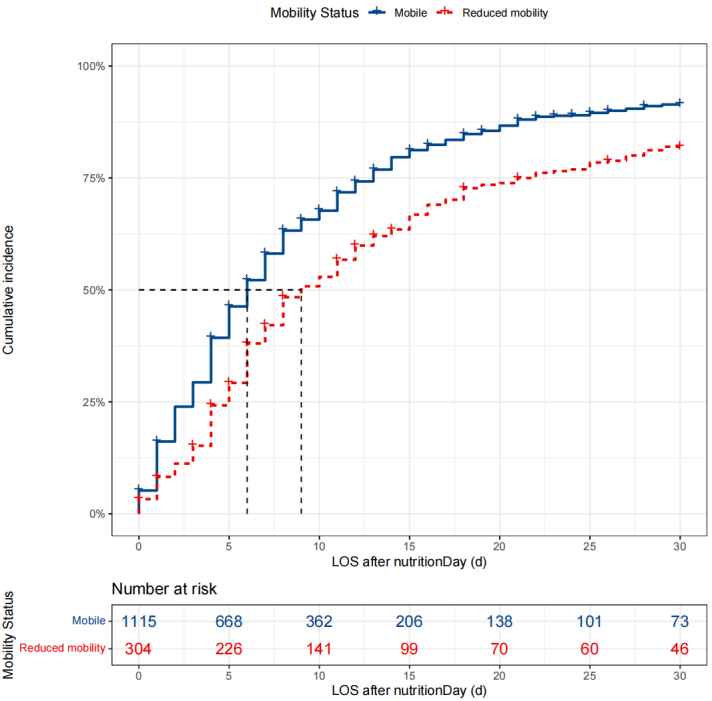 |
| LOS after nutritionDay: median (95% CI)  Patients with mobile vs. Patients with reduced mobility: 9 days (8-11) vs. 13 days (11-17), *p* < 0.01. | LOS after nutritionDay: median (95% CI)  Patients with mobile vs. Patients with reduced mobility: 6 days (6-7) vs. 9 days (8-11), *p* < 0.001. |

**Supplementary Figure S16.** Cumulative incidence of discharged home within 30 days after nutritionDay in patients with different nutritional interventions and meal eaten on the survey days (Sensitivity analyses without missing value and based on digestive disease of primary diagnosis). Differences in median (95% CI) LOS after nutritionDay between groups were tested using the log-rank test. LOS, length of hospital stay; CI, confidence interval. **(A)** Mobility status in patients with artificial nutrition, n = 696. **(B)** Mobility status in patients with dietary nutrition, n = 1721. **(C)** Mobility status in patients with completely eaten, n = 1240. **(D)** Mobility status in patients with incompletely eaten, n = 1632. **(E)** Mobility status in digestive disease patients with artificial nutrition, n = 645. **(F)** Mobility status in digestive disease patients with incompletely eaten, n = 1443.
